# Sleep apnea phenotyping and relationship to disease in a large clinical biobank

**DOI:** 10.1093/jamiaopen/ooab117

**Published:** 2022-01-11

**Authors:** Brian E Cade, Syed Moin Hassan, Hassan S Dashti, Melissa Kiernan, Milena K Pavlova, Susan Redline, Elizabeth W Karlson

**Affiliations:** Division of Sleep and Circadian Disorders, Brigham and Women’s Hospital, Boston, Massachusetts, USA; Division of Sleep Medicine, Harvard Medical School, Boston, Massachusetts, USA; Program in Medical and Population Genetics, Broad Institute, Cambridge, Massachusetts, USA; Division of Sleep and Circadian Disorders, Brigham and Women’s Hospital, Boston, Massachusetts, USA; Division of Sleep Medicine, Harvard Medical School, Boston, Massachusetts, USA; Division of Pulmonary Disease and Critical Care Medicine, University of Vermont, Burlington, Vermont, USA; Program in Medical and Population Genetics, Broad Institute, Cambridge, Massachusetts, USA; Center for Genomic Medicine, Massachusetts General Hospital and Harvard Medical School, Boston, Massachusetts, USA; Department of Anesthesia, Pain, and Critical Care Medicine, Massachusetts General Hospital, Boston, Massachusetts, USA; Division of Sleep and Circadian Disorders, Brigham and Women’s Hospital, Boston, Massachusetts, USA; NeuroCare Center for Sleep, Newton, Massachusetts, USA; Division of Sleep and Circadian Disorders, Brigham and Women’s Hospital, Boston, Massachusetts, USA; Division of Sleep Medicine, Harvard Medical School, Boston, Massachusetts, USA; Division of Sleep and Circadian Disorders, Brigham and Women’s Hospital, Boston, Massachusetts, USA; Division of Sleep Medicine, Harvard Medical School, Boston, Massachusetts, USA; Division of Pulmonary, Critical Care, and Sleep Medicine, Beth Israel Deaconess Medical Center, Boston, Massachusetts, USA; Center for Genomic Medicine, Massachusetts General Hospital and Harvard Medical School, Boston, Massachusetts, USA; Division of Rheumatology, Inflammation and Immunity, Brigham and Women's Hospital, Boston, Massachusetts, USA

**Keywords:** sleep-disordered breathing, sleep apnea, epidemiology, electronic health record, electronic medical record

## Abstract

**Objective:**

Sleep apnea is associated with a broad range of pathophysiology. While electronic health record (EHR) information has the potential for revealing relationships between sleep apnea and associated risk factors and outcomes, practical challenges hinder its use. Our objectives were to develop a sleep apnea phenotyping algorithm that improves the precision of EHR case/control information using natural language processing (NLP); identify novel associations between sleep apnea and comorbidities in a large clinical biobank; and investigate the relationship between polysomnography statistics and comorbid disease using NLP phenotyping.

**Materials and Methods:**

We performed clinical chart reviews on 300 participants putatively diagnosed with sleep apnea and applied International Classification of Sleep Disorders criteria to classify true cases and noncases. We evaluated 2 NLP and diagnosis code-only methods for their abilities to maximize phenotyping precision. The lead algorithm was used to identify incident and cross-sectional associations between sleep apnea and common comorbidities using 4876 NLP-defined sleep apnea cases and 3× matched controls.

**Results:**

The optimal NLP phenotyping strategy had improved model precision (≥0.943) compared to the use of one diagnosis code (≤0.733). Of the tested diseases, 170 disorders had significant incidence odds ratios (ORs) between cases and controls, 8 of which were confirmed using polysomnography (*n *= 4544), and 281 disorders had significant prevalence OR between sleep apnea cases versus controls, 41 of which were confirmed using polysomnography data.

**Discussion and Conclusion:**

An NLP-informed algorithm can improve the accuracy of case-control sleep apnea ascertainment and thus improve the performance of phenome-wide, genetic, and other EHR analyses of a highly prevalent disorder.

## Lay Summary

Sleep apnea is a common disease in which breathing partially or completely pauses during sleep, leading to less oxygen in the blood, repeated awakenings, and increased risk of developing multiple diseases. Current studies of sleep apnea often have relatively few participants due to the challenge of performing overnight sleep recordings. Electronic health record (EHR) billing code diagnoses of sleep apnea could be repurposed to increase the size of research studies, but the accuracy of the diagnoses is reduced. We developed a reusable algorithm that improves the accuracy of EHR sleep apnea diagnoses using natural language processing to extract information from clinical notes. As a proof of concept, we used the algorithm to identify hundreds of diseases that are increased among participants with sleep apnea compared to similar patients without sleep apnea. Many of these disease relationships with sleep apnea have not been previously recognized. This improved algorithm will help to accelerate future large-scale investigations of the causes and consequences of sleep apnea.

## BACKGROUND AND SIGNIFICANCE

Sleep apnea is a common disorder characterized by repetitive airway obstructions resulting in intermittent hypoxemia, sleep disruption, and multiple other physiological disturbances implicated in the pathogenesis of cardiovascular, metabolic, and neurological diseases.^1–17^ The estimated prevalence of sleep apnea in U.S. adults is 12%,^18^ although this varies by age and gender. Additional research in large, generalizable samples may lead to improved treatments for sleep apnea that lower the risk of developing multiple highly burdensome comorbidities.

Large-scale epidemiological research in electronic health record (EHR) biobanks enables multiple opportunities to accelerate research.^19–21^ Data collected as part of routine sleep clinic visits that would be financially and logistically challenging to collect prospectively may be efficiently repurposed for research questions, such as comprehensively identifying novel relationships with other diseases and improving the power of genetic analyses.^22–26^ However, certain challenges must be addressed. Early EHR analyses used International Classification of Disease (ICD) codes for sleep apnea phenotyping.^27^^,^^28^ ICD data are largely collected for clinical and billing purposes. ICD-derived disease diagnoses are often used when ruling out a given disease through a billed procedure such as a sleep examination whether or not the patient is found to have that condition. Modest diagnosis accuracy has been observed for several diseases, including a 33% positive predictive value for ICD-based rheumatoid arthritis phenotyping.^29^ Natural language processing (NLP) in conjunction with medical chart reviews can effectively improve phenotyping accuracy.^29–33^ Data from a sample of patients classified as true cases or true controls based on clinician validation (eg by manual record review using prespecified disease definitions) are extracted and linked to ICD-based diagnoses and other clinical information. Data from clinical notes within the EHR that improve classification accuracy in the validated set of charts are used to improve the diagnosis accuracy of other patients with ICD-based diagnoses. Extracting and processing free-text information is often addressed by using standardized vocabularies and medically oriented NLP tools.^34^^,^^35^ A second improvement has been to group similar ICD diagnoses into broader clinical categories of ≈1800 “PheCodes” to provide larger numbers of cases representing the disease of interest.^36^^,^^37^ Patients seen in an open healthcare system may seek care at institutions that are not part of the EHR system, and a third improvement is the use of a “data floor” with minimum healthcare utilization criteria to reduce misclassification of cases and controls due to incomplete EHR records.^31^ A fourth improvement for sleep apnea may result from extracting key values from clinical notes and available polysomnographic (PSG) summary reports, such as case/control status based on a disease-defining threshold for laboratory diagnosis of sleep apnea: the apnea-hypopnea index (AHI).

Here, we report the development of a validated NLP-informed phenotyping algorithm for sleep apnea in the Mass General Brigham (MGB) Biobank, a resource with over 120 000 participants.^38^^,^^39^ We compare the accuracy of this phenotyping algorithm to alternative models based on PheCodes and limited NLP,^40^ which are useful when medical charts or expert clinician review are not available. We constructed improved NLP phenotyping for comorbid diseases, providing an opportunity to examine the relationships between sleep apnea, PSG statistics, and other diseases. As a proof of concept, we report associations between the NLP-derived sleep apnea status prospective incidence and prevalence of multiple diseases. Many of these associations have limited or no previously tested association with sleep apnea.

## MATERIALS AND METHODS

Additional details are provided in the Supplemental Methods.

### Study sample

Participants contributed EHR and sample data and provided written research consent to the MGB Biobank.^38^^,^^39^ There were multiple analytical groups (Figure S1, Table  1). “Screen positive” sleep apnea cases were defined by ≥1 sleep apnea coded PheCode diagnoses (described below). We selected a random sample of 300 participants for detailed chart review in order to generate an algorithm to separate *bona fide* sleep apnea cases from false positive noncases (eg coded for billing purposes or otherwise erroneously). We selected 3× controls without PheCodes for sleep apnea or obstructive sleep apnea, matched to the NLP-defined sleep apnea cases based on age, sex, self-reported race/ethnicity, body mass index (BMI), and healthcare utilization using hospital encounters.^41^ We also examined 4544 participants with available polysomnography records irrespective of a sleep apnea diagnosis. The first sleep apnea diagnosis date or the date of the PSG recording was used to calculate the age of a participant. Controls were matched on birthdates relative to cases. The age of the first sleep apnea diagnosis for a given case was used as the age of a matched control. BMI was extracted from structured tables and from unstructured clinical notes using regular expressions. The 2 BMI measurements closest in time to the participant’s defined age were averaged together to calculate the participant’s defined BMI.

**Table 1.** Sample characteristics of samples used in different phases of the study

	All	Screen positive group	Chart review set	PheCAP cases	Polysomnography sample
*N*	100 616	15 741	300	4876	4544
Women, *N* (%)	56 910 (56.56)	6784 (43.10)	137 (45.67)	1887 (38.70)	2512 (55.28)
Mean age (IQR)	58.20 (25.65)	57.18 (18.19)	58.27 (18.94)	57.59 (16.87)	56.77 (24.60)
Mean BMI (IQR)	27.25 (7.78)	32.05 (9.66)	31.16 (10.48)	33.61 (10.15)	30.54 (9.73)
Race/ethnicity					
Asian, *N* (%)	2680 (2.66)	254 (1.62)	3 (1.00)	73 (1.50)	113 (2.49)
Black, *N* (%)	4930 (4.90)	941 (5.98)	18 (6.00)	356 (7.30)	585 (12.87)
Hispanic/Latino, *N* (%)	3778 (3.75)	590 (3.75)	15 (5.00)	188 (3.86)	386 (8.49)
White, *N* (%)	85 495 (84.97)	13 393 (85.08)	255 (85.00)	4084 (83.77)	2993 (65.87)
Other race/ethnicity, *N* (%)	3733 (3.71)	563 (3.58)	9 (3.00)	174 (3.57)	467 (10.28)
Language spoken					
English, *N* (%)	97 134 (96.54)	15 186 (96.47)	285 (95.00)	4711 (96.64)	3971 (87.39)
Spanish, *N* (%)	1456 (1.45)	220 (1.40)	4 (1.33)	76 (1.56)	442 (9.73)
Other languages, *N* (%)	2026 (2.01)	335 (2.13)	11 (3.67)	88 (1.80)	131 (2.88)

*Note*: “Screen positive” had one or more PheCode diagnoses for sleep apnea (327.3) or obstructive sleep apnea (327.32). The 300 participants in “Chart Review Set” were obtained from the Screen Positive Group and used to perform PheCAP phenotyping. “PheCAP Cases” were classified by lead PheCAP algorithm (PheCAP S_ICDNLP_ and NLP CUIs in Table  2). Age and BMI are presented as medians (interquartile range). All other fields, apart from sample size, are presented as total size (percentage). Age and BMI data were based on the first sleep apnea diagnosis date for PheCode cases, the last available visit date for PheCode controls, and the first available polysomnographic recording for the polysomnography sample.

*Abbreviations*: BMI: body mass index; IQR: interquartile range.

We employed a “data floor” to reduce the number of participants with minimal documentation and hence the likelihood of false negative associations in our open network healthcare setting.^20^ The sample was restricted to those with at least 2 clinical notes, 2 encounters associated with ICD diagnoses, and 3 separate PheCode diagnoses for any disease.

### Sleep apnea phenotype definitions

Following data floor filtering, screen-positive participants were selected based on one or more diagnoses with the sleep apnea PheCodes “327.3” (sleep apnea; ICD9 codes 327.2, 327.20, 327.29, 780.51, 780.53, 780.57; ICD 10 codes G47.3, G47.30, G47.39) and “327.32” (obstructive sleep apnea; ICD9 code 327.23; ICD10 code G47.33).^36^^,^^37^

### Clinician chart reviews

**Figure 1.**
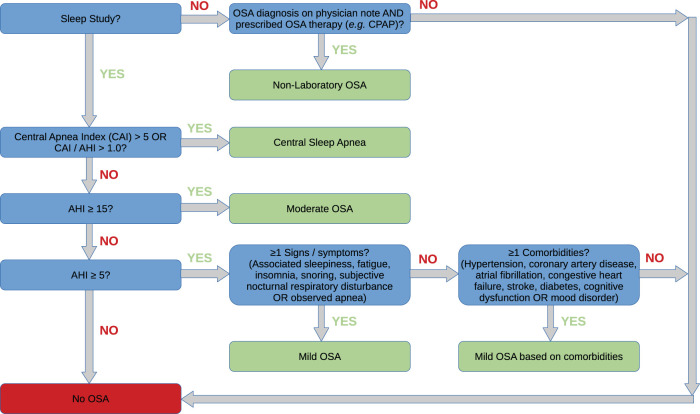
Sleep apnea chart review guidelines. Guidelines for adjudicating participants with ≥1 sleep apnea PheCode diagnoses were based on ICSD-3 criteria. Decision criteria in blue boxes resulted in either a true sleep apnea diagnosis (green boxes) or a noncase sleep apnea diagnosis.

We performed clinical chart reviews among the 300 ICD-screen positive participants, in order to create a gold standard set of sleep apnea cases and noncases for algorithm development. Sleep apnea case/noncase (ie false positive) classifications were adjudicated by 2 sleep clinicians (SR and SMH) and informed by ICSD-3 guidelines (Figure  1).^42^ Chart data from 97 screen negative participants were also used to assess predictive value of the negative screen. Sleep apnea case classification categories are marked in green in Figure  1, while noncase classification categories are marked in red. This approach outperformed 2 exploratory sleep apnea disease definition models that assigned participants with central sleep apnea (CSA) or all non-“moderate sleep apnea” classifications as noncases (data not shown). From the 300 chart review set, 180 (60%) of these results were used in the training set, and 120 (40%) were used in the validation set for PheCAP and other methods.

### Natural language processing

We extracted NLP terms that mapped to Concept Unique Identifiers (CUIs) from the Unified Medical Language System using cTAKES, and counted the instances of each nonnegated CUI term per note.^34^^,^^35^ We used 2 NLP-based algorithm development approaches. (1) PheCAP distinguishes true cases from noncases (ie negative in chart reviews despite one or more ICD codes) based on the presence of common terms extracted from the literature.^31^ (2) Multimodal Automated Phenotyping (MAP) omits chart reviews and supplements ICD codes with the count of their exact matches located within clinical notes (eg count of “obstructive sleep apnea” phrases).^40^

Sleep apnea candidate CUI terms were obtained using the surrogate-assisted feature extraction (SAFE) method from 7 internet-derived disease review resources (Table S1)^33^^,^^43^ in order to select NLP concepts commonly recognized with sleep apnea and therefore more likely to generalize to other populations. We also constructed a composite term based on the cumulative count of 6 sleep apnea-related procedures and 2 NLP terms described in Table S2 that we term the “Joint CPAP CUI/Procedure Term.”

### PheCAP phenotype classification

We used PheCAP to test algorithms to classify sleep apnea case/noncase status in the chart review training and validation sets.^31^ We tested 20 separate models to identify the optimal PheCAP settings (Table S3). PheCAP allows for flexible surrogate “silver standards” of a phenotype that aid in classification. We tested multiple surrogate combinations of S_ICD_ (the number of phenotype PheCode diagnoses of a given patient), S_NLP_ (the cumulative number of NLP CUI disease terms (eg “sleep apnea”) seen across clinical notes for a given patient), and S_ICDNLP_ (a combined count of the 2 terms). We tested the inclusion and exclusion of demographic (age, sex, and self-reported race/ethnicity) plus BMI, and PheCAP NLP terms. We further tested the final optimized PheCAP model to ask whether forcing case status for participants with diagnostic polysomnography criteria for sleep apnea (AHI ≥ 15)^42^ and/or the joint continuous positive airway pressure ventilation (CPAP) CUI/procedure term from clinical notes and/or from PSG reports would improve overall model performance (*n* = 13 cases, 7 noncases with measures available). The overall level of healthcare utilization has been shown to bias NLP analyses.^31^ We therefore adjusted for the number of encounters with an ICD code for each participant in each PheCAP algorithm model.

### Statistical analyses

Our primary measures of algorithm performance for PheCAP models, compared with PheCAP definitions, and the MAP model were the area under the receiver operator characteristic curve (AUC) and precision (Table  2). Five additional statistics are provided in Table S3.

**Table 2.** Chart review performance of alternative sleep apnea phenotyping algorithms

Method	Training recall (sensitivity)	Training precision (PPV)	Training negative predictive value	Training AUC	Validation recall (sensitivity)	Validation precision	Validation negative predictive value	Validation AUC
≥1 PheCode	1.000	0.689	NA	NA	1.000	0.733	NA	NA
≥2 PheCodes	0.823	0.836	0.621	NA	0.795	0.805	0.455	NA
MAP NLP CUIs	0.774	0.850	0.582	0.819	0.727	0.831	0.442	0.786
PheCAP S_ICDNLP_ and NLP CUIs	0.427	0.981	0.437	0.893	0.375	0.943	0.353	0.832
PheCAP S_ICD_	0.387	0.941	0.411	0.790	0.341	0.938	0.341	0.756
PheCAP S_NLP_	0.331	0.911	0.385	0.820	0.250	0.917	0.313	0.753
PheCAP S_ICDNLP_	0.331	0.911	0.385	0.822	0.250	0.917	0.313	0.754
PheCAP S_ICDNLP_, Demographics, and NLP CUIs	0.403	0.980	0.426	0.892	0.352	0.939	0.345	0.830
PheCAP S_ICDNLP_ and NLP CUIs plus AHI and CPAP	0.411	0.981	0.430	0.904	0.443	0.951	0.380	0.858

*Note*: A total of 300 chart reviews were performed for participants with one or more sleep apnea PheCode codings. Therefore, certain PheCode-only rows lack negative predictive values by definition. Of the 300 chart reviews, 180 (60%) of these results were used in the training set, and 120 (40%) were used in the validation set. Results for the best performing PheCAP model are shown as “PheCAP S_ICDNLP_ and NLP CUIs,” along with chart review performance for PheCode-only definitions using a minimum of 1 and 2 PheCode instances to define a case and a more basic NLP algorithm using MAP. The performance of PheCAP surrogate-only models is shown next (“PheCAP S_ICD_,” “PheCAP S_NLP_,” “PheCAP S_ICDNLP_”) and is followed by the predictive performance using demographic parameters exclusively. Reduced performance was observed when including demographics and the lead PheCAP model (“PheCAP S_ICDNLP_, Demographics, and NLP CUIs”). Additional modest performance gains were obtained by forcing case status for participants with separately extracted AHI and/or continuous positive airway pressure (joint CPAP CUI/procedure term) evidence. Full results for all models are presented in Table S5. Recall (sensitivity) = true positives/(true positives + false negatives). Precision (Positive Predictive Value) = true positives/(true positives + false positives); Negative Predictive Value = true negatives/(true negatives + false negatives).

*Abbreviations:* AHI: apnea-hypopnea index; AUC: area under the curve; CPAP: continuous positive airway pressure ventilation; CUIs: concept unique identifiers; MAP: multimodal automated phenotyping; NLP: natural language processing.

Chi-square analyses examined the prevalence and incidence of comorbid PheCodes in PheCAP cases based on the best performing PheCAP algorithm compared to matched controls. We considered 527 PheCodes with a minimum MGB Biobank case prevalence of 1%. An incident diagnosis was defined as the first diagnosis for a comorbidity occurring at least one year after the first sleep apnea diagnosis. Participants with a prior diagnosis were excluded. Analyses considered combined sex and sex-stratified strata. Bonferroni corrections adjusted for the combined count of overall, female, and male analyses.

Logistic regression was used to analyze potential associations between PSG statistics and cross-sectional or incident comorbidities that were significantly associated with PheCAP sleep apnea status by adjusting for age and BMI at the time of the first available PSG recording, sex, and self-reported race/ethnicity. Phenotypes were then rank-normalized to account for any nonnormality in these residual values. We analyzed 2 PSG summary statistics: the AHI using 3% criteria and the percentage of the sleep episode with oxyhemoglobin saturation <88% (Per88). Tests were performed for PheCodes that were significantly associated with sleep apnea PheCAP status in combined-sex analyses. Bonferroni adjustments considered the combined count of AHI and Per88 calculations.

## RESULTS

### Sample characteristics

Sample characteristics are listed in Table  1. From the initial sample of 115 124 participants, 108 597 participants were retained after removing children or those without suitable criteria for the data floor. The final sample size was 100 616 after removing participants with unknown age, sex, and/or BMI values. Within this sample, 15,741 participants had ≥1 PheCode diagnoses for sleep apnea or obstructive sleep apnea, yielding 15.6% prevalence. Data from 397 randomly selected participants were used for chart review, including data from 300 participants with ≥1 sleep apnea PheCode diagnoses (and used in the algorithm validations) and data from 97 sleep apnea PheCode controls (to query for false negative PheCode diagnoses). From this sample, data from 180 participants with adjudicated case/control status (60% of those with a positive sleep apnea PheCode diagnosis) were used in training and data from 120 participants with adjudicated case/control status were used in validation. Three of the 97 participants without an ICD diagnosis for sleep apnea were determined to have sleep apnea based on chart reviews.

### PheCAP algorithm construction and performance

The 7 articles used for SAFE yielded 1072 nonnegated NLP concepts (CUI terms^34^) that were seen in at least one article (eg “PSG (Polysomnography) [Diagnostic Procedure]”). A total of 130 terms were present in a majority of the articles and in at least one clinical note of ≥5% of participants with a sleep apnea PheCode diagnosis and were retained for analysis.

We tested 20 alternative PheCAP models using the 130 CUI terms and demographic and BMI data to identify the optimal tunable parameters (Tables  2 and S3). We present representative algorithms from PheCodes, PheCAP, and MAP in Table  2 based on chart review classification of cases/noncases using ICSD-3 guidelines and including sleep apnea and physician notes supported by prescribed therapy (Figure  1). The lead PheCAP model with the maximum AUC values (“PheCAP S_ICDNLP_ and NLP CUIs” in Table  2) was based on cases and noncases classified as in the Figure  1 guidelines, combined counts of PheCode codings and equivalent PheCode NLP phrases (the S_ICDNLP_ surrogate model), and additional NLP terms. Better AUC performance was observed when demographic and BMI data were excluded. Nevertheless, the average age and BMI and the percentage of males were all higher in the final ascertained PheCAP case sample compared to the PheCode-only screen positive group (Table  1). Final beta coefficients for NLP terms in the tested PheCAP models are provided in Table S4. The lead model included nonzero coefficients for the intercept, the number of clinical encounters, CUI C0199451 (CPAP, initiation and management), and the combined S_ICDNLP_ silver standard surrogate term of sleep apnea PheCode counts, C0037315 (sleep apnea), and C05200679 (obstructive sleep apnea syndrome).The PheCAP algorithm is designed to optimize precision. The lead PheCAP model had improved precision in chart reviews of participants with at least one PheCode-based diagnosis coding date compared to PheCode-only counts (≥0.943 vs ≤0.733; Table  2). Modest predictive improvements were observed when forcing PheCAP controls with an observed AHI ≥ 15 and/or an observed joint CPAP CUI/procedure term to be PheCAP cases (precision ≥ 0.951).

### Associations between sleep apnea and comorbidities

We used the lead PheCAP model (ie PheCAP S_ICDNLP_ and NLP CUIs without the AHI or the joint CPAP CUI/procedure term to increase the generalizability of our findings) to define sleep apnea cases, inform the selection of 3× matched controls, and test the prevalence and incidence of comorbidities. We reused the nonsleep apnea NLP terms generated as a by-product of sleep apnea PheCAP phenotyping to generate NLP-informed case/control phenotyping using MAP and data from all of the MGB Biobank participants with a minimum data floor. We then tested the incidence of new comorbidities, defined by considering a first comorbidity diagnosis that occurred at least a year after the first sleep apnea diagnosis (Tables  3 and S5). Out of 527 tested PheCodes, 170 PheCodes had significant odds ratios (ORs) in combined-sex and/or sex-stratified analyses following Bonferroni correction. Hypersomnia and restless legs syndrome (RLS) had the highest odds ratio point estimates, likely due to participants being followed in sleep clinics. Lead disease associations reflected a range of pathobiology, including hypertensive heart disease, hypoglycemia, dysthymic disorder, and dementias. Diseases with significantly reduced incidence odds ratios included secondary malignancy of bone and non-Hodgkin’s lymphoma. In sex-stratified analyses (Table S5), 76 comorbidities had significant odds ratios considering women with and without sleep apnea, while 111 comorbidities had significant odds ratios considering men with and without sleep apnea. While many disorders had relatively similar odds ratio estimates in both analyses, several disorders had higher odds ratio point estimates and/or nonoverlapping odds ratio confidence interval estimates among participants with sleep apnea in one sex versus the other sex. PheCodes with the largest incidence odds ratio differences between women and men for nonsleep disorders are provided in Figure S2. Notably, the chronic pulmonary heart disease odds ratio was higher in women (OR 4.17, 95% CI, 2.84–6.14) compared to men (OR 1.80, 95% CI, 1.32–2.44; *P* for sex interaction = 7.15 × 10^−4^). Gout also had higher odds ratio estimates in women (OR 3.27, 95% CI, 2.34–4.56) compared to men (OR 1.36, 95% CI, 1.13–1.63; *P* for sex interaction = 6.61 × 10^−6^). Obesity had a higher odds ratio estimate in men (OR 3.05, 95% CI, 2.63–3.53) compared to women (OR 1.50, 95% CI, 1.25–1.81; *P* for sex interaction = 1.24 × 10^−4^).

**Table 3.** Incident disease enrichment among sleep apnea cases

PheCode	Translation	Odds ratio	Incidence in SA cases	Incidence in matched controls
327.1	Hypersomnia	16.38 (11.55–23.24)	4.71	0.30
327.71	Restless legs syndrome	5.55 (4.34–7.09)	4.19	0.78
263	Other nutritional deficiency	4.26 (3.38–5.35)	4.09	0.99
428.4	Heart failure with preserved ejection fraction (Diastolic heart failure)	3.75 (3.07–4.58)	5.02	1.39
278.11	Morbid obesity	3.72 (3.20–4.32)	9.60	2.78
401.21	Hypertensive heart disease	3.05 (2.52–3.69)	4.96	1.68
278.4	Abnormal weight gain	2.94 (2.44–3.54)	5.22	1.84
327	Sleep disorders	2.91 (2.35–3.59)	4.03	1.42
470	Septal deviations/turbinate hypertrophy	2.87 (1.97–4.19)	1.21	0.42
472	Chronic pharyngitis and nasopharyngitis	2.69 (2.00–3.61)	1.90	0.72
1002	Symptoms concerning nutrition, metabolism, and development	2.64 (2.27–3.07)	7.54	3.00
415.2	Chronic pulmonary heart disease	2.50 (1.97–3.16)	2.93	1.19
291.8	Alteration of consciousness	2.49 (2.02–3.08)	3.60	1.47
251.1	Hypoglycemia	2.47 (1.92–3.19)	2.45	1.01
313.1	Attention-deficit hyperactivity disorder	2.45 (1.87–3.22)	2.17	0.89
306.9	Tension headache	2.40 (1.63–3.52)	1.06	0.44
276.6	Fluid overload	2.34 (1.93–2.83)	4.26	1.87
300.4	Dysthymic disorder	2.33 (1.92–2.83)	4.38	1.93
428.2	Heart failure not otherwise specified	2.32 (1.93–2.78)	4.90	2.17
798.1	Chronic fatigue syndrome	2.32 (1.81–2.97)	2.50	1.09
296.22	Major depressive disorder	2.31 (2.07–2.56)	17.10	8.21
290.1	Dementias	2.27 (1.73–2.98)	2.09	0.93
1013	Asphyxia and hypoxemia	2.26 (1.86–2.74)	4.26	1.93
539	Bariatric surgery	2.25 (1.99–2.53)	11.52	5.47
278.1	Obesity	2.25 (2.00–2.52)	19.00	9.46

*Note*: An incident diagnosis was defined as the first diagnosis for a potential comorbidity occurring at least one year after the first diagnosis date for sleep apnea. Otherwise, participants with prior diagnoses were excluded. Sample sizes will therefore vary by PheCode. Totally, 527 PheCodes with ≥1% overall prevalence were tested. Controls were matched for age, sex, BMI, population, and healthcare utilization. It was found that 170 nonredundant PheCodes were significantly associated following Bonferroni correction. Lead results are shown here. Complete results, including sex-stratified results and sample sizes, can be found in Table S5.

*Abbreviations*: SA: sleep apnea.

We calculated the cross-sectional prevalence of 527 PheCode diagnoses among sleep apnea PheCAP cases and matched controls. Of this, 281 nonredundant PheCodes had significant odds ratios in combined-sex and/or sex-stratified analyses after Bonferroni adjustment (Tables  4 and S6). Morbid obesity had the highest odds ratio point estimate for any nonsleep disorder, followed by heart failure with preserved ejection fraction. The most significantly enriched PheCodes in cross-sectional analyses included cardiac, pulmonary, and multiple mental health and mood disorders. Secondary malignancy of bone was significantly associated and had a reduced prevalence among sleep apnea cases (OR 0.37, 95% CI, 0.26–0.54). In sex-stratified analyses (Table S6), 174 disorders had significant ORs considering women with and without sleep apnea; 219 disorders had significant odds ratios considering men with and without sleep apnea. PheCodes with the largest absolute OR point estimate differences between women and men for nonsleep disorders are shown in Figure S3. Three heart conditions had higher, nonoverlapping OR estimates in women compared to men: chronic pulmonary heart disease, congestive heart failure not otherwise specified, and heart failure not otherwise specified (*P* for sex interaction ≤ 4.83 × 10^−3^).

**Table 4.** Cross-sectional disease enrichment among sleep apnea cases

PheCode	Translation	Odds ratio	Prevalence in sleep apnea cases	Prevalence in matched controls
327.1	Hypersomnia	21.52 (15.95–29.02)	7.92	0.40
327.71	Restless legs syndrome	6.77 (5.55–8.27)	7.18	1.13
278.11	Morbid obesity	5.56 (5.02–6.16)	23.94	5.36
327	Sleep disorders	4.61 (4.01–5.29)	11.63	2.78
428.4	Heart failure with preserved ejection fraction (Diastolic heart failure)	4.45 (3.79–5.23)	8.41	2.02
415.2	Chronic pulmonary heart disease	3.99 (3.35–4.75)	6.77	1.79
278.1	Obesity	3.67 (3.42–3.93)	52.06	22.84
512.9	Other dyspnea	3.64 (3.35–3.94)	32.81	11.84
263	Other nutritional deficiency	3.54 (2.97–4.23)	6.13	1.81
1013	Asphyxia and hypoxemia	3.28 (2.84–3.78)	9.15	2.98
470	Septal deviations/turbinate hypertrophy	3.13 (2.46–3.99)	3.02	0.98
512.7	Shortness of breath	3.08 (2.85–3.33)	34.54	14.61
401.21	Hypertensive heart disease	2.92 (2.50–3.41)	7.22	2.60
509.1	Respiratory failure	2.89 (2.43–3.43)	5.87	2.11
296.22	Major depressive disorder	2.86 (2.65–3.10)	32.48	14.38
428.1	Congestive heart failure (CHF) not otherwise specified	2.80 (2.55–3.09)	19.26	7.84
276.6	Fluid overload	2.79 (2.40–3.25)	7.34	2.76
291.8	Alteration of consciousness	2.74 (2.31–3.24)	5.97	2.27
278.4	Abnormal weight gain	2.70 (2.39–3.05)	11.80	4.72
539	Bariatric surgery	2.61 (2.36–2.88)	17.31	7.43
290.3	Other persistent mental disorders due to conditions classified elsewhere	2.58 (2.13–3.13)	4.33	1.72
505	Other pulmonary inflammation or edema	2.57 (2.15–3.08)	4.98	2.00
496	Chronic airway obstruction	2.53 (2.25–2.86)	11.38	4.82
313.1	Attention-deficit hyperactivity disorder	2.51 (2.08–3.02)	4.56	1.87
798.1	Chronic fatigue syndrome	2.51 (2.05–3.07)	3.90	1.59

*Note*: Totally, 527 PheCodes with ≥1% overall prevalence were tested. Controls were matched for age, sex, BMI, population, and healthcare utilization. Of the tested PheCodes, 281 nonredundant PheCodes had significantly different cross-sectional prevalence between PheCAP-defined cases and matched controls in combined-sex and/or sex-stratified analyses. Lead results are shown here. Complete results, including sex-stratified results, can be found in Table S6.

### Validation of associated comorbidities using polysomnography

Finally, we performed similar incident and cross-sectional comorbidity analyses in 4544 participants with available polysomnography. We tested the AHI using 3% desaturation criteria and the percentage of the sleep recording with oxyhemoglobin saturation under 88% (Per88). Eight largely cardiopulmonary and circulatory diseases were significantly associated with PSG measures in analyses of incident cases, including hypertensive heart disease (*P* = 7.62 × 10^−9^; Table S7). Forty-one diseases had significant cross-sectional associations after Bonferroni adjustment (Table S8). Several cardiopulmonary diseases were associated, including asphyxia and hypoxemia (*P* = 2.10 × 10^−41^) and chronic pulmonary heart disease (*P* = 1.99 × 10^−20^). The lowest *P* values for 37 of these PheCodes were observed when analyzing Per88 (Figure S4). Of the 17 diseases that were highly associated with Per88, 10 diseases (*P* < 1 × 10^−10^) were not nominally associated with AHI (*P* > .05).

## DISCUSSION

In this study, we constructed an improved sleep apnea phenotyping algorithm that addresses the limitations of ICD codings within the EHR by using NLP and controlling for healthcare utilization to improve precision. This algorithm considered CPAP usage and can be applied to important analyses examining the causes and consequences of sleep apnea. We applied this algorithm as a proof of principle in a phenome-wide analysis that identified multiple disorders with elevated incidence and prevalence in patients with sleep apnea compared to matched controls. The phenotyping of the nonsleep disorders was also improved using NLP, and to our knowledge, most disorders have never previously been examined in the context of sleep apnea. The association between sleep apnea and the incidence and/or prevalence of several of these disorders was confirmed using polysomnography, despite a modest sample size and single point-in-time polysomnography data.

The PheCAP algorithm was designed to optimize phenotype precision (Tables  2 and S3), which is particularly useful for genetic analyses and prioritizing the selection of true cases with high certainty. The precision of the validation sample improved from 0.733 when using a single diagnosis date (≥1 PheCodes) to 0.943 when applying the PheCAP algorithm. CPAP is the most frequent medical treatment for sleep apnea and, with few exceptions, is used almost exclusively in outpatient settings for treating sleep apnea. The CPAP usage NLP term identified by PheCAP would likely generalize to other healthcare systems. Inclusion of AHI from polysomnography and CPT codes or other structured data signifying the use of CPAP improves phenotyping precision slightly (0.943–0.951). Additional improvements gained using the PheCAP procedure include the use of a “data floor” to exclude participants with sparse EHR documentation and adjustment for healthcare utilization to control for biases,^41^ which were likely to have improved the precision of the ≥1 PheCodes and all other algorithms. Putative cases can be restricted to those with multiple ICD diagnosis coding dates to improve precision in situations where access to text and/or procedural data is impossible.

We systematically examined the potential relationships between sleep apnea cases, matched controls, and comorbid diseases by leveraging improvements in the diagnostic accuracy of comorbidities using NLP.^20^^,^^30^^,^^40^ The majority of tested diseases (170 incident PheCodes and 281 cross-sectional PheCodes out of 527 tested PheCodes) had significantly different incidence and/or prevalence rates between sleep apnea cases and controls following Bonferroni corrections (Tables S5 and S6). Given the known associations of sleep apnea with multiple metabolic, cardiovascular, and neurocognitive morbidities,^1^ this is not surprising. These data highlight the role of sleep apnea as a risk factor for a broad range of diseases. Unexpectedly, patients with sleep apnea were at lower risk for incident diagnoses for non-Hodgkin’s lymphoma and secondary malignancy of bone, with similar directionality in the cross-sectional results. We will attempt to replicate these results in future studies as these could be due to practice patterns in our system. Further work is needed to understand the pathophysiological mechanisms between sleep apnea and these diseases, the relative contributions of sleep apnea compared to competing risk factors for these diseases, and whether certain sleep apnea subtypes and groups of comorbidities have potential statistical relationships, which may aid in improved patient risk stratification and more personalized treatment strategies.

Personalized treatment may involve different gender-specific strategies. A number of comorbid diseases had odds ratio estimates that diverged in sex-stratified analyses (Tables S5 and S6, Figures S1 and S2). There are well-described gender differences in the physiology of sleep apnea, with men generally having more hypoxemia and women having more arousals^44^—factors that may influence propensity for future diseases. A portion of the differential odds ratios between men and women for specific diseases may be due to differences in sleep apnea subtypes.^44–46^ Notable PheCodes that have higher odds ratios of incidence in women include chronic pulmonary heart disease, gout, and congestive heart failure not otherwise specified.

Multiple sleep disorders are often observed in the same patients. Other sleep disorders, including RLS, had higher odds ratios in cross-sectional analyses (Table  4). The RLS association may be due in part to an increased awareness of sleep clinicians who may screen for other sleep disorders when examining patients suspected of having sleep apnea. RLS prevalence is increased among patients with sleep apnea versus controls, and RLS symptoms are reduced after treatment for sleep apnea.^47^^,^^48^ We could not completely disentangle the effects of central versus obstructive sleep apnea, as 90% of the participants originally diagnosed with CSA were also diagnosed with obstructive sleep apnea. “Cardiac defibrillator in situ” and “delirium due to conditions classified elsewhere” were significantly associated in a sensitivity analysis considering patients with a CSA diagnosis versus matched controls (*P* ≤ 8.40 × 10^−4^). The nonoverlapping odds ratio estimates were higher in the CSA diagnosis group compared to the remainder of the sample without a prior CSA diagnosis. Future work is needed to determine whether these odds ratio differences are due to CSA-specific effects, and whether comorbid sleep disorders have additive effects that may contribute to an increased prevalence and/or incidence of comorbid disease.

Most of the lead associations between polysomographic traits and comorbidities were based on a measure of low overnight oxygen saturation during sleep (Per88), in contrast to the more commonly used AHI (Tables S9 and S10, Figure S4). This is consistent with prior single disease reports^49–51^ but has not been systematically evaluated to our knowledge. Hypoxemia measures have been the bases of our most significant genetic associations with sleep-disordered breathing to date.^24^ Ten of the 17 diseases that were highly associated with Per88 (*P* < 1 × 10^−10^) in cross-sectional analyses were not nominally associated with AHI (*P* > .05; Table S8, Figure S4), indicating that a readily available PSG summary measure is more significantly associated with dozens of comorbidities compared to the AHI. Additional associations may be observed in the future using more specific measures such as the hypoxic burden.^52^ The AHI (a count of the number of breathing pauses per hour of sleep) is increasingly recognized as a heterogeneous marker, resulting in a wide variety of stresses due to differences in durations and severity of individual breathing pauses that comprise the AHI.^46^ Increased AHI was associated with reduced likelihood of cross-sectionally ascertained bariatric surgery, essential hypertension, migraine and, notably, insomnia. The latter association may reflect the common occurrence of co-morbid insomnia with sleep apnea^53^ or the increased likelihood of sleep disorder recognition once a patient is referred to a sleep specialist. The strength of a disease’s association with measures of disrupted sleep versus hypoxemia may provide insights into potential pathophysiological connections for future study.

### Strengths and weaknesses

Strengths of this study include applying advanced NLP methods to large-scale sleep phenotyping for the first time, to our knowledge. Careful consideration of comorbidity phenotyping and adjustment for healthcare utilization^41^ increases our confidence in the association of sleep apnea with the increased prevalence of hundreds of disorders, using a phenome-wide approach. We validated these associations with several disorders using polysomnography. Measures of hypoxemia may be more sensitive to the risk of certain disorders compared to the AHI.

While our algorithm may conceivably not generalize to other environments, similar portable algorithms have been demonstrated for other phenotypes.^29^^,^^54^ Moreover, the SAFE algorithm was designed to extract common concepts from background literature,^33^ reducing the risk of overfitting. The CPAP term that remained predictive following cross-validated LASSO regression represents a first-line therapy used in clinical sleep laboratories. We will attempt to replicate and extend our findings in other diverse biobanks in future studies.

## CONCLUSION

We developed an advanced sleep apnea clinical phenotyping algorithm that was able to increase the precision of EHR data by leveraging NLP and identified several novel cross-sectional and incident associations between sleep apnea and other diseases. Despite their challenges, large-scale EHR analyses have provided important insights into the biology of disease.^55^^,^^56^ EHR analyses of sleep apnea will be an attractive, pragmatic pathway for advancing our understanding of this important disorder at an unprecedented scale.

## FUNDING

Brian E. Cade is supported by grants from the National Institutes of Health (R01-HL153805, K01-HL135405, and R03-HL154284) and the American Thoracic Society Foundation. Susan Redline is supported by grants from the National Institutes of Health (R35-HL135818). Elizabeth W. Karlson is supported by grants from the National Institutes of Health (U01-HG008685 and P30 AR070253).

## AUTHOR CONTRIBUTIONS

The authors contributed to the paper as follows: Conception and design: BEC, SMH, SR, and EWK.

Data acquisition: BEC, SMH, HSD, MK, MKP, SR, and EWK.

Analysis: BEC.

Interpretation, draft and review, and final approval: all authors.

BEC and EWK had full access to the study data and take responsibility for the integrity of the data and accuracy of analyses.

## SUPPLEMENTARY MATERIAL

Supplementary material is available at *JAMIA Open* online.

## Supplementary Material

ooab117_Supplementary_Data

## Data Availability

The data underlying this article are available in the article and in its online supplementary material.
