# Supplementary material for: Sleep apnea phenotyping and relationship to disease in a large clinical biobank
Source: JAMIA Open. 2022 Jan 11;5(1):ooab117. doi: 10.1093/jamiaopen/ooab117 (PMC8826997; doi:10.1093/jamiaopen/ooab117)

Figure S1. Study population overview

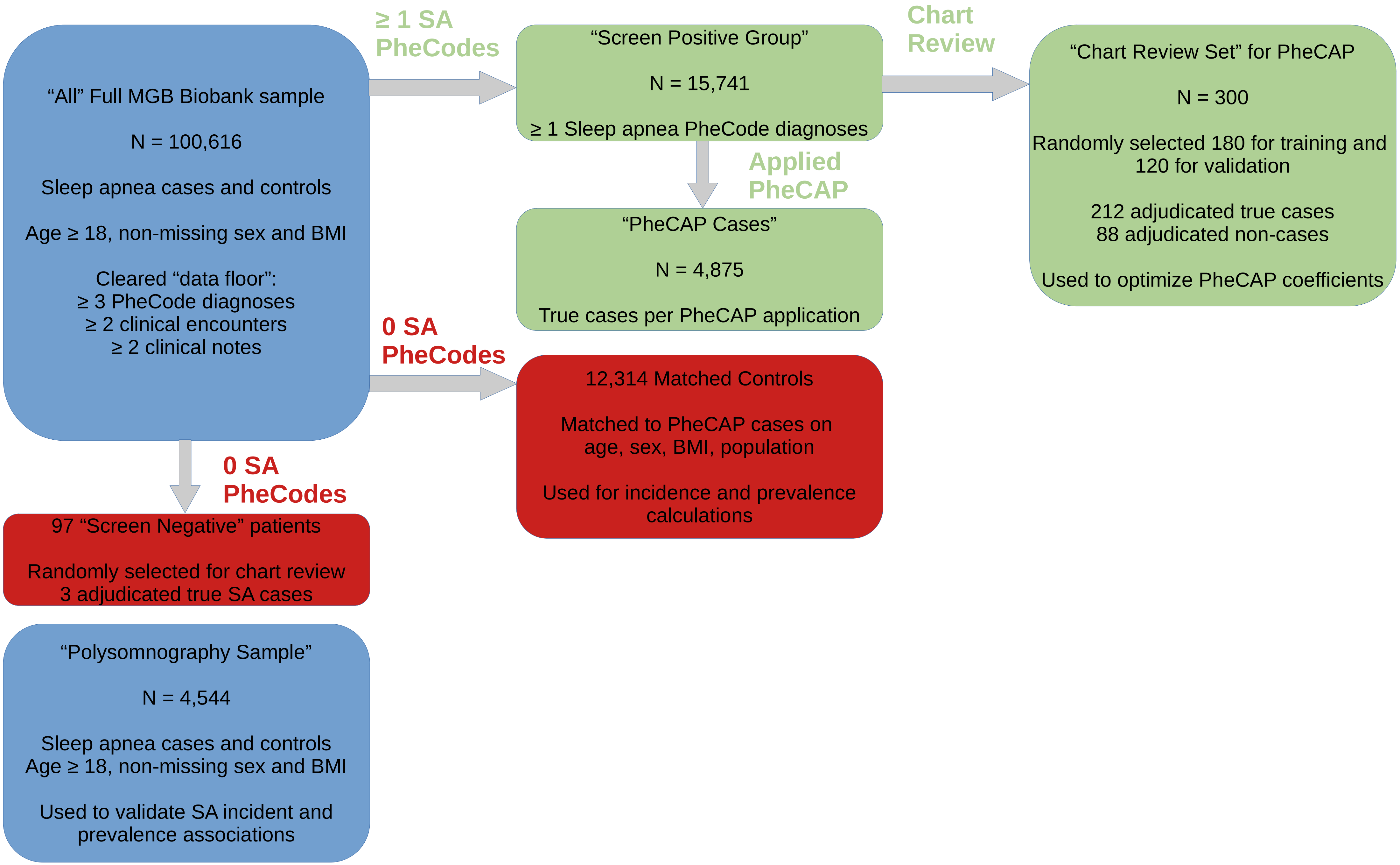

Figure S2. Lead sex−stratified incident non−sleep disorder associations

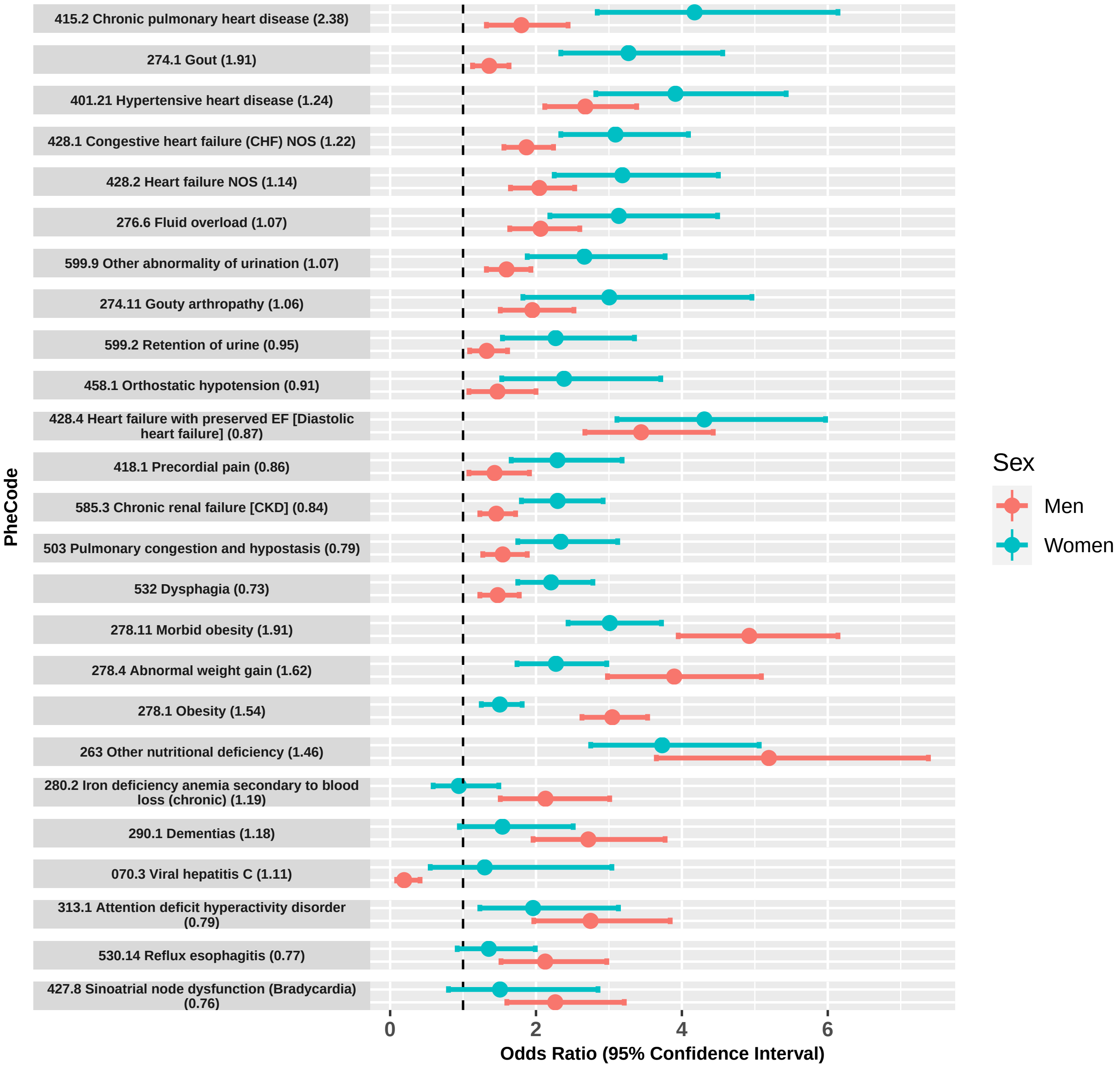

Figure S3. Lead sex−stratified cross−sectional non−sleep disorder associations

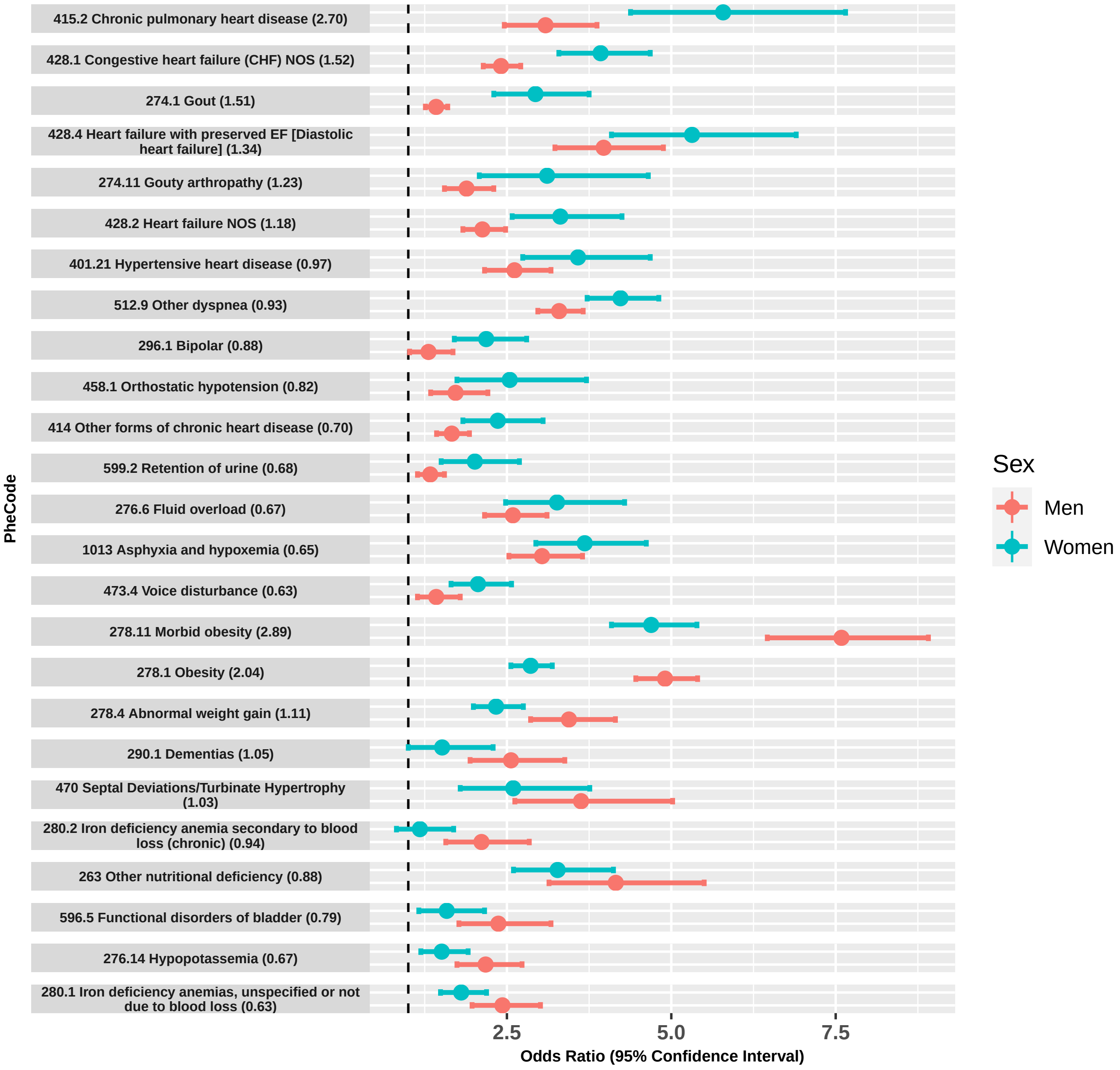

Figure S4. AHI 3% and Per88 PheCode cross−sectional association p−values

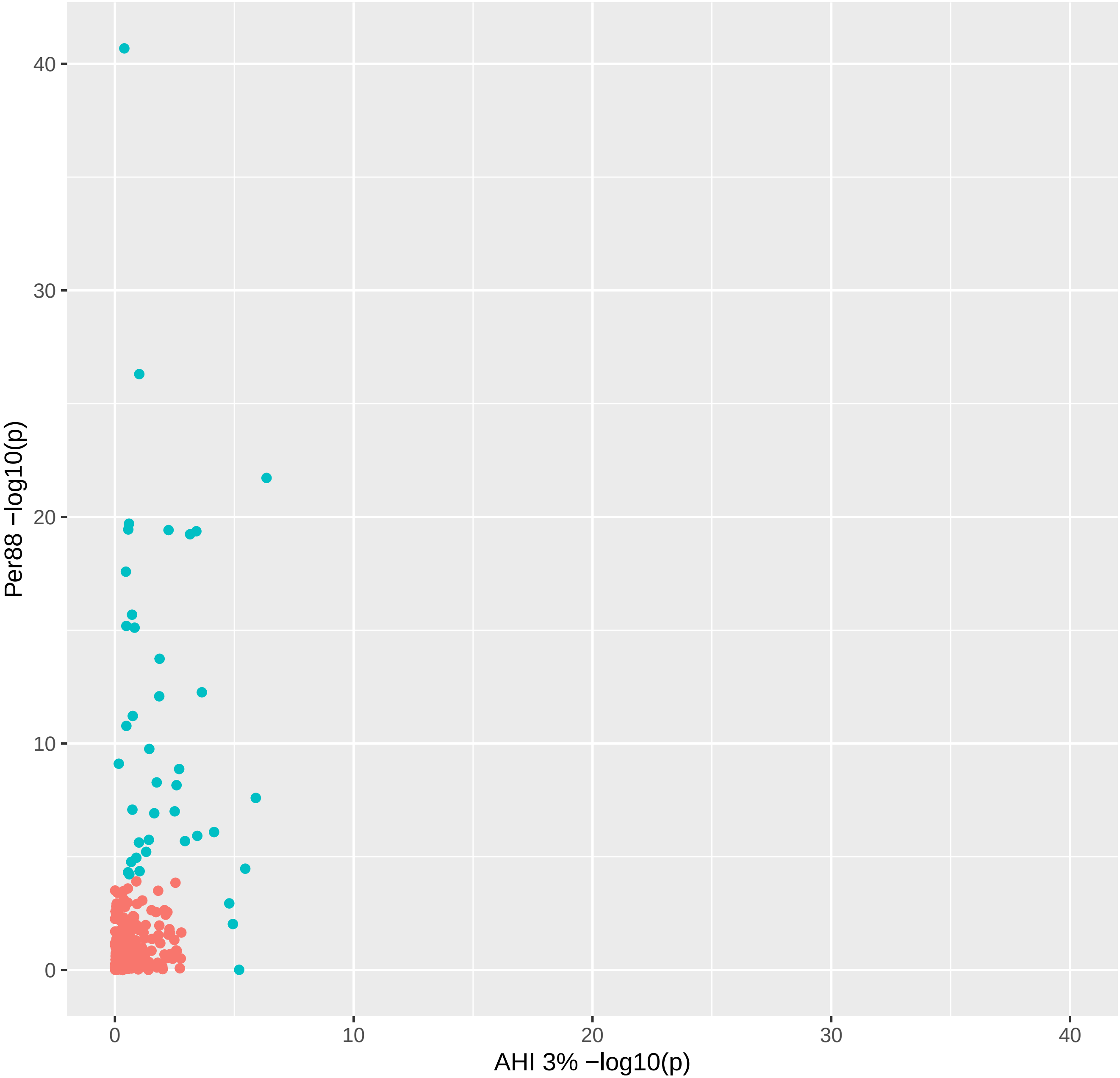

Supplement: ooab117_Supplementary_Data [file ooab117_Supplementary_Data.zip › Cade-EHR-sleep-apnea-algorithm-supplemental-figures-20211006.pdf]
