# Supplementary material for: Sleep apnea phenotyping and relationship to disease in a large clinical biobank
Source: JAMIA Open. 2022 Jan 11;5(1):ooab117. doi: 10.1093/jamiaopen/ooab117 (PMC8826997; doi:10.1093/jamiaopen/ooab117)
Supplement: ooab117_Supplementary_Data [file ooab117_Supplementary_Data.zip › Cade-EHR-sleep-apnea-algorithm-supplemental-methods.pdf]

**Sleep Apnea Phenotyping and Relationship to Disease in a Large Clinical Biobank**

## SUPPLEMENTAL METHODS

### Study sample

Study participants contributed EHR and sample data to the MGB Biobank [1,2], which is a data warehouse comprised of patient records from multiple hospitals. Data include date-stamped EHR clinical notes, ICD9 and ICD10 diagnoses, procedures, inpatient and outpatient encounter information, medication data, demographics, and genetic data on a subset of participants. A large portion of the data are structured and have been curated using the i2b2 platform [3]. MGB Biobank data are derived from the larger MGB Research Patient Data Registry, which is comprised of clinical data collected from 1986 to the present. Participants provided written consent for their data to be used for epidemiological, genomic, and other research. Our sample was comprised of participants from four hospitals: Brigham and Women's Hospital, Faulkner Hospital, Massachusetts General Hospital, and Spaulding Rehabilitation Hospital. Data from 115,124 participants were obtained, prior to data cleaning. The demographics of this data set are broadly representative of the MGB system, with a slight female bias (56.6%). European or "white" race was indicated by 85.4% of the participants, either by self-report or as recorded by a healthcare provider, in line with the population of eastern Massachusetts. A subset of 419 participants with available genotyping and "other" or unknown populations were assigned continental (and admixed Hispanic/Latino American) populations based on principal component analyses in conjunction with 1000 Genomes Project and Human Genome Diversity Project data [4,5]. English was the first language of 96.5% of the sample.

The study sample was comprised of multiple analytical groups used in different analyses (**Figure S1, Table 1**). Most data were obtained from the Biobank sample and included putative sleep apnea cases and controls, defined by at least one sleep apnea coded PheCode diagnosis. From the putative "screen positive" sleep apnea PheCode cases, we selected a random sample of 300 participants for detailed chart review in order to generate an algorithm to separate *bona fide* sleep apnea cases from false positive sleep apnea PheCode cases (e.g. coded only for billing purposes). Once we determined the NLP-based sleep apnea cases from the entire set of putative sleep apnea cases, we then selected 3× sleep apnea controls matched on age (± 5 years), sex, self-reported population, BMI (± 5 units), and one of eight bins of healthcare utilization using the sum of hospital encounters [6]. Controls were matched on birthdates relative to cases. The age of the first sleep apnea diagnosis for a given case was used as the age of a matched control. We examined 4,544 participants with available polysomography in order to test whether PSG statistics would also be associated with cross-sectional and incident comorbidity disease status. These participants were derived from the MGB Research Patient Data Registry, which includes participants from outside of the MGB Biobank [2,7].

We employed a “data floor” to reduce the number of participants with minimal visit information and hence the likelihood of false negative associations in our open network healthcare setting [8]. Therefore, we restricted analyses to participants with at least two clinical notes and two encounters associated with ICD diagnoses. Diagnoses were binned into PheCode classes across all ICD9 and ICD10 categories [9,10]. The sample was restricted to those with three separate PheCode diagnoses (*e.g.* the same diagnosis on three separate days or multiple PheCode diagnosis classifications from the same day).

## Clinician chart reviews

We performed clinical chart reviews among the 300 ICD-screen positive participants, in order to create a gold standard set of sleep apnea cases and non-cases for algorithm development. We performed clinical chart reviews among 97 screen negative participants to assess predictive value of the negative screen. Sleep apnea case / non-case (i.e. false positive) classifications were adjudicated by two sleep clinicians and informed by ICSD-3 guidelines (**Figure 1**) [11]. Chart data from 97 screen negative participants were also used to assess predictive value of the negative screen. We investigated inclusion and exclusion of central sleep apnea in the NLP phenotyping, however we omitted distinct analysis of the 327.31 central sleep apnea PheCode. There was a high overlap with other sleep apnea diagnoses (as 90% of the 802 patients with a CSA 327.31 PheCode diagnosis also had a 327.3 and/or a 327.32 PheCode diagnosis). Sleep apnea case classification categories are marked in green in Figure 1, while sleep apnea non-case classification categories are marked in red. This approach outperformed two additional exploratory sleep apnea disease definition models that assigned participants with central sleep apnea or all non-”moderate sleep apnea” classifications as non-cases (data not shown). The first 20 classifications were independently adjudicated by two sleep clinicians in order to identify any technical issues and to ensure that ICSD guidelines were operationalized appropriately. Patients could be classified as having sleep apnea due to a) a diagnosis on a clinical note with prescribed therapy (*e.g.* continuous positive airway pressure [CPAP]) in the absence of a recorded AHI value, termed “non-laboratory OSA”; b) “moderate OSA” with an AHI ≥ 15 visible within the clinical note and/or polysomnography report; c) “mild OSA” with a documented AHI ≥ 5 and < 15 with associated sleepiness, fatigue, insomnia, snoring, subjective nocturnal respiratory disturbance, or observed apnea; or d) mild OSA consisting of a documented AHI ≥ 5 and < 15 with associated comorbidities (including hypertension, coronary artery disease, atrial fibrillation, congestive heart failure, stroke, Type 2 diabetes, cognitive dysfunction, or mood disorder). Participants with a documented central apnea index ≥ 5 or a central apnea index / AHI ratio ≥ 1 were classified as having central sleep apnea. Three of the eight participants classified as having central sleep apnea also had OSA PheCode diagnoses and were judged to have treatment emergent sleep apnea. Therefore these participants were

also classified as having OSA. Patients with an OSA diagnosis on a clinical note but with no evidence of CPAP or other treatment or a corroborating AHI level were classified as non-cases. From the 300 chart review set, 180 (60%) of these results were used in the training set, and 120 (40%) were used in the validation set.

**Natural language processing**

We extracted NLP terms that mapped to Concept Unique Identifiers (CUIs) from the National Library of Medicine Unified Medical Language System (UMLS) using the cTAKES 4.0 default clinical pipeline, and counted the instances of each non-negated CUI term per note [12,13]. Term counts were aggregated across four analyzed categories of notes (discharge summaries, pre-EPIC progress notes, EPIC progress notes, and EPIC clinic visit notes). We constructed NLP-based definitions using two approaches that use a subset of these CUI terms. PheCAP is an approach to distinguish true cases (*i.e.* positive in chart reviews) from non-cases (*i.e.* negative in chart reviews despite one or more ICD codings) based on the presence of common terms extracted from the literature [14]. Multimodal Automated Phenotyping (MAP) is an approach without chart reviews that supplements ICD codings with the count of their exact text matches located within clinical notes (*e.g.* "obstructive sleep apnea") [15].

Sleep apnea candidate NLP CUI terms were obtained using the surrogate-assisted feature extraction method (SAFE) [16] from seven internet-derived disease review resources: American Thoracic Society Patient Series, Mayo Clinic, Medline, Medscape, Merck Manuals, Nature Reviews Disease Primers, and Wikipedia (**Table S1**) and extracted using MetaMap [17]. 130 CUI terms that were observed in a majority of the internet-derived disease review resources and were present in the clinical notes of ≥5% patients with sleep apnea were retained for algorithm testing. We further used data on specific treatments for sleep apnea- CPAP, polysomnography with CPAP, and sleep apnea treatments with lower usage (e.g. palatopharyngoplasty). We also manually constructed a composite term based on the cumulative count of six relevant procedures and two NLP terms described in **Table S2** that we term the "Joint CPAP CUI/Procedure Term".

We used NLP CUI terms to improve the phenotyping of non-sleep apnea diseases. Each disease was based on a PheCode grouping of one or more ICD codes. The text phrases for each PheCode were obtained from the PheWAS Catalog site ( https://phewascatalog.org/phecodes ). Text from individual ICD code descriptions was obtained from the UMLS MRCONZO database. Cumulative counts of relevant UMLS CUI terms for each PheCode and its composite ICD codes were summed across all clinical notes for each participant for analysis in MAP.

**PheCAP and MAP phenotype classification**

We used PheCAP methods to test algorithms to classify sleep apnea case/non-case status in the chart review training and validation sets [14]. This algorithm allows for flexible definitions of "silver standards" of a phenotype and tunable parameters to aid in NLP CUI term selection. Multiple phenotype surrogates can be used to define these silver standards. $S_{ICD}$ denotes the number of phenotype ICD (or, in this analysis, PheCode) diagnoses of a given patient, $S_{NLP}$ denotes the cumulative number of NLP CUI terms (*e.g.* the phrase "sleep apnea") seen across clinical notes for a given patient, and $S_{ICDNLP}$ indicates a joint measure of the two terms. Each term includes tunable upper and lower limits to define silver-standard cases and controls. We optimized each parameter choice based on the best performing area under the curve (AUC) values for randomly chosen splits of the data when fixing other parameter choices. We used 1 – 5 $S_{ICD}$ counts of diagnosis dates, 4 – 10 $S_{NLP}$ counts of CUI terms (C0037315 "sleep apnea" and C0520679 "obstructive sleep apnea syndrome"), and 4 – 15 $S_{ICDNLP}$ combinations of the $S_{ICD}$ and $S_{NLP}$ terms. CUI terms were selected in PheCAP using adaptive LASSO regression with 5-fold cross validation, as this method yielded the highest random-split AUC criteria compared to other available choices. PheCAP evaluates surrogates in groups, yielding different levels of model performance that can vary by phenotype. We tested five surrogate models that use alternative strategies to combine the coded diagnosis (ICD) and clinical text phrase (NLP) information: $S_{ICD}$, $S_{NLP}$, $S_{ICDNLP}$, $S_{ICD}$ + $S_{NLP}$, and $S_{ICD}$ + $S_{NLP}$ + $S_{ICDNLP}$. We tested four combinations of variables within PheCAP: 1) surrogate terms alone; 2) surrogate and demographic terms including age, sex, BMI, and self-reported race/ethnicity; 3) surrogate and NLP terms; and 4) surrogate, demographic, and NLP CUI terms. We tested 20 separate models to identify the optimal PheCAP settings (**Table S3**). We further tested the final optimized PheCAP model to ask whether forcing case status for participants with diagnostic polysomnography criteria for sleep apnea (AHI ≥15) and/or the joint CPAP CUI/procedure term would improve overall model performance. AHIs were obtained from PSG reports and extracted from the clinical notes using custom regular expressions and visually verified. The overall level of healthcare utilization has been shown to bias NLP analyses [14]. We therefore adjusted for the number of encounters with an ICD code (dx_enc) for each participant in each PheCAP algorithm model. Data from 180 participants (60%) were used for PheCAP training and data from 40% of participants were used for PheCAP validation.

MAP analyses were performed for all PheCodes using the same source of NLP terms and healthcare utilization statistics as the PheCAP analyses (**Table S3**). While PheCAP performance in sleep apnea chart reviews was superior to MAP performance (**Table 2**), MAP remains useful for improving the accuracy of secondary analyses compared to ICD or PheCode counts alone, without the time and clinical expertise needed for detailed chart reviews. We used MAP to generate participant case/control status for non-sleep apnea PheCodes with a minimum prevalence of 1% in order to test

the prevalence and incidence of other diseases among participants with and without sleep apnea. We copied the equivalent participant IDs used during PheCAP training and validation for comparisons with MAP, as MAP does not use training and validation datasets. PheCode-only models are similarly divided based on PheCAP training and validation participant IDs.

## Statistical analyses

Our primary measures of PheCAP performance were the area under the receiver operator characteristic curve (AUC) and precision (**Table 2**). AUC statistics are unavailable for direct PheCAP counts (which do not produce model predictions). We also calculated accuracy, negative predictive value, recall, specificity, and F1 scores (**Table S3**).

Chi-square analyses of the prevalence and incidence of comorbid PheCodes in PheCAP cases compared to matched controls and supercontrols were performed in R using the epiR package. We considered PheCodes with a minimum case prevalence of 1% across the entire MGB Biobank sample. An incident diagnosis was defined as the first diagnosis for a potential comorbidity occurring at least one year after the first diagnosis date for sleep apnea. Otherwise, participants with prior diagnoses were excluded and classified as existing. Sex-interaction tests were based on generalized linear models in R by considering the interaction term of the logistic regression formula (comorbidity status ~ sleep apnea status * sex + age + BMI + race/ethnicity).

Logistic regression was used to analyze potential associations between PSG statistics and cross-sectional or incident comorbidities by adjusting for age and BMI at the time of the first available PSG recording, biological sex, and self-reported race/ethnicity. Phenotypes were first adjusted for covariates then rank-normalized to account for any non-normality in these residual values. All p-values are based on rank-normalized traits, while betas and standard errors were calculated based on non-transformed phenotypes to improve statistical interpretation. We analyzed two statistics that were readily available within the PSG summary reports. In addition to the apnea-hypopnea index using 3% criteria, we analyzed the percentage of the sleep episode with oxyhemoglobin saturation <88% (Per88) due to past reports where hypoxemia measures were more associated with adverse outcomes compared to the AHI (e.g. [18–20]).

**SUPPLEMENTAL REFERENCES**


1. Weiss ST, Shin MS. Infrastructure for Personalized Medicine at Partners HealthCare. *J Pers Med*. 2016;6(1):13. doi:10.3390/jpm6010013

2. Karlson EW, Boutin NT, Hoffnagle AG, Allen NL. Building the Partners HealthCare Biobank at Partners Personalized Medicine: Informed Consent, Return of Research Results, Recruitment Lessons and Operational Considerations. *J Med*. 2016;6(1).

3. Murphy SN, Weber G, Mendis M, et al. Serving the enterprise and beyond with informatics for integrating biology and the bedside (i2b2). *J Am Med Inform Assoc JAMIA*. 2010;17(2):124-130. doi:10.1136/jamia.2009.000893

4. Wang C, Zhan X, Liang L, Abecasis GR, Lin X. Improved ancestry estimation for both genotyping and sequencing data using projection procrustes analysis and genotype imputation. *Am J Hum Genet*. 2015;96(6):926-937. doi:PMCID: PMC4457959.

5. Cade BE, Chen H, Stilp AM, et al. Associations of Variants In the Hexokinase 1 and Interleukin 18 Receptor Regions with Oxyhemoglobin Saturation During Sleep. *PLoS Genet*. 2019;15(4):e1007739.

6. Castro VM, Apperson WK, Gainer VS, et al. Evaluation of matched control algorithms in EHR-based phenotyping studies: a case study of inflammatory bowel disease comorbidities. *J Biomed Inform*. 2014;52:105-111. doi:10.1016/j.jbi.2014.08.012

7. Weiss ST, Shin MS. Infrastructure for Personalized Medicine at Partners HealthCare. *J Pers Med*. 2016;6(1):13. doi:10.3390/jpm6010013

8. Robinson JR, Wei W-Q, Roden DM, Denny JC. Defining Phenotypes from Clinical Data to Drive Genomic Research. *Annu Rev Biomed Data Sci*. 2018;1(1):69-92. doi:10.1146/annurev-biodatasci-080917-013335

9. Denny JC, Ritchie MD, Basford MA, et al. PheWAS: demonstrating the feasibility of a phenome-wide scan to discover gene-disease associations. *Bioinforma Oxf Engl*. 2010;26(9):1205-1210. doi:10.1093/bioinformatics/btq126

10. Denny JC, Bastarache L, Ritchie MD, et al. Systematic comparison of phenome-wide association study of electronic medical record data and genome-wide association study data. *Nat Biotechnol*. 2013;31(12):1102-1110. doi:10.1038/nbt.2749

11. Sateia MJ. International classification of sleep disorders-third edition: highlights and modifications. *Chest*. 2014;146(5):1387-1394. doi:10.1378/chest.14-0970

12. Bodenreider O. The Unified Medical Language System (UMLS): integrating biomedical terminology. *Nucleic Acids Res*. 2004;32(Database issue):D267-270. doi:10.1093/nar/gkh061

13. Savova GK, Masanz JJ, Ogren PV, et al. Mayo clinical Text Analysis and Knowledge Extraction System (cTAKES): architecture, component evaluation and applications. *J Am Med Inform Assoc JAMIA*. 2010;17(5):507-513. doi:10.1136/jamia.2009.001560

14. Zhang Y, Cai T, Yu S, et al. High-throughput phenotyping with electronic medical record data using a common semi-supervised approach (PheCAP). *Nat Protoc*. 2019;14(12):3426-3444. doi:10.1038/s41596-019-0227-6

15. Liao KP, Sun J, Cai TA, et al. High-throughput multimodal automated phenotyping (MAP) with application to PheWAS. *J Am Med Inform Assoc*. Published online August 7, 2019. doi:10.1093/jamia/ocz066

16. Yu S, Chakrabortty A, Liao KP, et al. Surrogate-assisted feature extraction for high-throughput phenotyping. *J Am Med Inform Assoc JAMIA*. 2017;24(e1):e143-e149. doi:10.1093/jamia/ocw135

17. Aronson AR, Lang F-M. An overview of MetaMap: historical perspective and recent advances. *J Am Med Inform Assoc JAMIA*. 2010;17(3):229-236. doi:10.1136/jamia.2009.002733

18. Kendzerska T, Gershon AS, Hawker G, Leung RS, Tomlinson G. Obstructive sleep apnea and risk of cardiovascular events and all-cause mortality: a decade-long historical cohort study. *PLoS Med*. 2014;11(2):e1001599. doi:PMCID: PMC3913558.

19. Gellen B, Canouï-Poitrine F, Boyer L, et al. Apnea-hypopnea and desaturations in heart failure with reduced ejection fraction: Are we aiming at the right target? *Int J Cardiol*. 2016;203:1022-1028.

20. Minville C, Hilleret M-N, Tamisier R, et al. Nonalcoholic fatty liver disease, nocturnal hypoxia, and endothelial function in patients with sleep apnea. *Chest*. 2014;145(3):525-533. doi:10.1378/chest.13-0938
