# Supplementary material for: Sleep apnea phenotyping and relationship to disease in a large clinical biobank
Source: JAMIA Open. 2022 Jan 11;5(1):ooab117. doi: 10.1093/jamiaopen/ooab117 (PMC8826997; doi:10.1093/jamiaopen/ooab117)
Supplement: ooab117_Supplementary_Data [file ooab117_Supplementary_Data.zip › Cade-EHR-sleep-apnea-algorithm-table-s1.doc]

**Table S1. URLs for the SAFE term sources**

These seven sources were used to extract common terms to describe clinical aspects of sleep apnea that were then extracted from the clinical notes for use in PheCAP. Multiple documents from a single source were combined together for processing in MetaMap. The web pages were accessed 8/1/2019.

| **Name** | **URL** |
| --- | --- |
| American Thoracic Society Patient Series | https://www.thoracic.org/patients/patient-resources/resources/obstructive-sleep-apnea-in-adults.pdf |
| American Thoracic Society Patient Series | https://www.thoracic.org/patients/patient-resources/resources/cpap-for-osa.pdf |
| American Thoracic Society Patient Series | https://www.thoracic.org/patients/patient-resources/resources/oral-appliances-sleep-apnea.pdf |
| American Thoracic Society Patient Series | https://www.thoracic.org/patients/patient-resources/resources/other-therapies-for-sleep-apnea.pdf |
| Mayo Clinic | https://www.mayoclinic.org/diseases-conditions/obstructive-sleep-apnea/symptoms-causes/syc-20352090 |
| Mayo Clinic | https://www.mayoclinic.org/diseases-conditions/central-sleep-apnea/symptoms-causes/syc-20352109 |
| Medline | https://medlineplus.gov/ency/article/000811.htm |
| Medline | https://medlineplus.gov/ency/article/003997.htm |
| Medscape | https://emedicine.medscape.com/article/295807-overview |
| Medscape | https://emedicine.medscape.com/article/304967-overview |
| Merck Manuals | https://www.merckmanuals.com/professional/pulmonary-disorders/sleep-apnea/obstructive-sleep-apnea |
| Merck Manuals | https://www.merckmanuals.com/professional/pulmonary-disorders/sleep-apnea/central-sleep-apnea |
| Nature Reviews Disease Primers | https://www.nature.com/articles/nrdp201515 |
| Wikipedia | https://en.wikipedia.org/wiki/Obstructive_sleep_apnea |
| Wikipedia | https://en.wikipedia.org/wiki/Central_sleep_apnea |
