# Supplementary material for: Sleep apnea phenotyping and relationship to disease in a large clinical biobank
Source: JAMIA Open. 2022 Jan 11;5(1):ooab117. doi: 10.1093/jamiaopen/ooab117 (PMC8826997; doi:10.1093/jamiaopen/ooab117)
Supplement: ooab117_Supplementary_Data [file ooab117_Supplementary_Data.zip › Cade-EHR-sleep-apnea-algorithm-table-s2.doc]

**Table S2. Sleep apnea intervention procedure and clinical note terms**

We constructed an omnibus field of cumulative sleep apnea treatment CUIs and procedures and tested MAP and PheCAP models with and without this term. This composite term is referred to in other tables as the “Joint CPAP CUI/Procedure Term”. We used the six available terms from the structured procedures data that are related to CPAP or other sleep apnea interventions (e.g. BiPAP, mandibular advancement devices, uvulopalatopharyngoplasty). We also searched for 135 CUIs potentially related to sleep apnea interventions (as extracted from the UMLS MRCONZO database). Three of the 135 CUIs were located and present in at least one note for >0.1% of the total sample.

| **Term type** | **Code** | **Description** |
| --- | --- | --- |
| Procedure | ICD10 5A09357 | Assistance with Respiratory Ventilation, Less than 24 Consecutive Hours, Continuous Positive Airway Pressure |
| Procedure | ICD9 93.90 | Continuous positive airway pressure [CPAP] |
| Procedure | CPT 94660 | Continuous positive airway pressure ventilation (CPAP), initiation and management |
| Procedure | EPIC 46000005 | Cpap Ventilation Cpap Initiation&Mgmt HC |
| Procedure | CPT 42145 | Palatopharyngoplasty (eg, uvulopalatopharyngoplasty, uvulopharyngoplasty) |
| Procedure | EPIC 74000014 | Polysom 6/>yrs Sleep W/Cpap 4/> Addl Param Attnd HC |
| Procedure | CPT 95811 | Polysomnography, sleep staging with 4 or more additional parameters of sleep, with initiation of continuous positive airway pressure therapy or bilevel ventilation, attended by a technologist |
| Clinical note term | C0021778 | Positive pressure ventilation |
| Clinical note term | C0199451 | Continuous positive airway pressure |
| Clinical note term | C1535578 | Palatopharyngoplasty [e.g. uvulopalatopharyngoplasty, uvulopharyngoplasty] |
