# Supplementary material for: Sleep apnea phenotyping and relationship to disease in a large clinical biobank
Source: JAMIA Open. 2022 Jan 11;5(1):ooab117. doi: 10.1093/jamiaopen/ooab117 (PMC8826997; doi:10.1093/jamiaopen/ooab117)
Supplement: ooab117_Supplementary_Data [file ooab117_Supplementary_Data.zip › Cade-EHR-sleep-apnea-algorithm-table-s4.doc]

**Table S4. PheCAP terms and coefficient weights**

Terms selected by SAFE and surrogate terms for the lead PheCAP model (3. Surrogates and CUIs; Sicdnlp) are shown. Non-zero coefficients indicate that a given term was included in the final PheCAP LASSO model.

| **Term** | **Coefficient** | **Translation** |
| --- | --- | --- |
| (Intercept) | -1.4379 | Intercept |
| encounter_dates_with_icds | -0.3833 | patient_dxenct |
| cui_C0003578 | 0.0000 | Apnea |
| cui_C0037315 | 0.0000 | Sleep apnea |
| cui_C0199451 | 0.7630 | Continuous positive airway pressure ventilation (CPAP), initiation and management |
| cui_C0235546 | 0.0000 | Hypopnea |
| cui_C0520679 | 0.0000 | Obstructive sleep apnea syndrome |
| sleep_apnea_phecode_joint_unique_dates | 0.0000 | Unique PheCode dates |
| sleep_apnea_phecode_joint_unique_dates&cui_C0037315&cui_C0520679 | 1.0690 | Unique PheCode dates & sleep apnea & obstructive sleep apnea syndrome |
