# Supplementary material for: Sleep apnea phenotyping and relationship to disease in a large clinical biobank
Source: JAMIA Open. 2022 Jan 11;5(1):ooab117. doi: 10.1093/jamiaopen/ooab117 (PMC8826997; doi:10.1093/jamiaopen/ooab117)
Supplement: ooab117_Supplementary_Data [file ooab117_Supplementary_Data.zip › Cade-EHR-sleep-apnea-algorithm-table-s7.doc]

**Table S7. Polysomnograpic Associations with Incident PheCode Case Status**

The first PSG recordings from 4,544 participants (with any sleep apnea status) were extracted. All 166 PheCodes that were significant in the combined-sex incident Table S5 analysis were tested. Multimodal Automated Phenotyping (MAP) was performed similarly to the Table S5 sample, and case/control status was recalibrated based on the cut-offs in the MGB Biobank sample. Incident cases were defined as occurring at least one year after the first PSG recording. Existing cases were set to null, resulting in different sample sizes for each PheCode. Logistic regression analyses examined AHI and Per88 (percent of the sleep recording under 88% oxyhemoglobin saturation) and PheCode case status by adjusting for the age and BMI at the time of the PSG, sex, and self-reported population. Clinical encounter count was adjusted for during the MAP phenotype construction. All traits were adjusted for covariates and then rank-normalized. *: Cleared Bonferroni p-value correction.

| **PheCode** | **Translation** | **Min P** | **N** | **Per88 Beta** | **Per88 SE** | **Per88 P** | **AHI Beta** | **AHI SE** | **AHI P** |
| --- | --- | --- | --- | --- | --- | --- | --- | --- | --- |
| 401.21 | Hypertensive heart disease | 7.62E-09* | 4,424 | 0.055 | 0.009 | 7.62E-09 | 0.012 | 0.005 | 2.09E-02 |
| 458.9 | Hypotension NOS | 2.04E-08* | 4,403 | 0.056 | 0.010 | 2.04E-08 | 0.011 | 0.006 | 6.37E-02 |
| 285 | Other anemias | 3.03E-08* | 3,907 | 0.046 | 0.008 | 3.03E-08 | 0.006 | 0.005 | 1.56E-01 |
| 276.6 | Fluid overload | 2.05E-07* | 4,410 | 0.052 | 0.010 | 2.05E-07 | 0.005 | 0.006 | 4.04E-01 |
| 1015 | Effects of other external causes | 1.95E-05* | 4,271 | 0.036 | 0.008 | 1.95E-05 | 0.008 | 0.004 | 7.66E-02 |
| 496 | Chronic airway obstruction | 2.23E-05* | 4,272 | 0.065 | 0.015 | 2.23E-05 | -0.002 | 0.010 | 8.43E-01 |
| 428.2 | Heart failure NOS | 7.80E-05* | 4,396 | 0.057 | 0.014 | 7.80E-05 | 0.023 | 0.008 | 5.93E-03 |
| 949 | Allergies, other | 1.45E-04* | 4,289 | 0.005 | 0.011 | 6.36E-01 | -0.020 | 0.005 | 1.45E-04 |
| 505 | Other pulmonary inflamation or edema | 1.87E-04 | 4,481 | 0.052 | 0.014 | 1.87E-04 | 0.010 | 0.008 | 2.40E-01 |
| 503 | Pulmonary congestion and hypostasis | 2.32E-04 | 4,360 | 0.042 | 0.011 | 2.32E-04 | 0.014 | 0.006 | 1.64E-02 |
| 480 | Pneumonia | 5.24E-04 | 4,180 | 0.031 | 0.009 | 5.24E-04 | 0.004 | 0.004 | 3.73E-01 |
| 764 | Sciatica | 1.69E-03 | 4,237 | 0.032 | 0.010 | 1.69E-03 | 0.007 | 0.005 | 1.83E-01 |
| 782.3 | Edema | 1.77E-03 | 4,062 | 0.032 | 0.010 | 1.77E-03 | -0.011 | 0.005 | 4.45E-02 |
| 599.8 | Other symptoms involving urinary system | 2.56E-03 | 4,491 | 0.041 | 0.014 | 2.56E-03 | 0.000 | 0.008 | 9.71E-01 |
| 741.4 | Joint effusions | 2.80E-03 | 4,148 | 0.026 | 0.009 | 2.80E-03 | 0.001 | 0.004 | 8.17E-01 |
| 539 | Bariatric surgery | 4.32E-03 | 4,160 | -0.016 | 0.014 | 2.58E-01 | -0.016 | 0.006 | 4.32E-03 |
| 501 | Pneumonitis due to inhalation of food or vomitus | 4.43E-03 | 4,522 | 0.048 | 0.017 | 4.43E-03 | 0.011 | 0.010 | 2.42E-01 |
| 459.1 | Hemorrhage NOS | 4.83E-03 | 4,486 | 0.039 | 0.014 | 4.83E-03 | -0.007 | 0.008 | 3.52E-01 |
| 426.21 | First degree AV block | 4.84E-03 | 4,486 | 0.021 | 0.017 | 2.11E-01 | 0.022 | 0.008 | 4.84E-03 |
| 586 | Other disorders of the kidney and ureters | 6.57E-03 | 4,301 | 0.035 | 0.013 | 6.57E-03 | 0.005 | 0.007 | 4.65E-01 |
| 729 | Other disorders of soft tissues | 6.63E-03 | 4,317 | 0.020 | 0.007 | 6.63E-03 | -0.005 | 0.004 | 1.41E-01 |
| 788 | Syncope and collapse | 7.01E-03 | 4,169 | 0.033 | 0.012 | 7.01E-03 | -0.008 | 0.007 | 2.05E-01 |
| 338.1 | Acute pain | 7.78E-03 | 4,033 | 0.008 | 0.007 | 2.16E-01 | -0.008 | 0.003 | 7.78E-03 |
| 250.42 | Other abnormal glucose | 8.02E-03 | 4,136 | 0.031 | 0.012 | 8.02E-03 | 0.004 | 0.006 | 5.41E-01 |
| 418.1 | Precordial pain | 8.36E-03 | 4,421 | -0.013 | 0.022 | 5.39E-01 | -0.024 | 0.009 | 8.36E-03 |
| 530.14 | Reflux esophagitis | 9.07E-03 | 4,360 | -0.082 | 0.031 | 9.07E-03 | -0.014 | 0.009 | 1.46E-01 |
| 519.8 | Other diseases of respiratory system, NEC | 9.11E-03 | 4,452 | 0.029 | 0.011 | 9.11E-03 | 0.006 | 0.006 | 2.87E-01 |
| 1013 | Asphyxia and hypoxemia | 1.03E-02 | 4,311 | 0.034 | 0.013 | 1.03E-02 | -0.015 | 0.007 | 2.89E-02 |
| 797 | Shock | 1.26E-02 | 4,527 | 0.038 | 0.015 | 1.26E-02 | -0.001 | 0.008 | 8.60E-01 |
| 414 | Other forms of chronic heart disease | 1.35E-02 | 4,444 | 0.036 | 0.014 | 1.35E-02 | 0.016 | 0.007 | 3.72E-02 |
| 286.7 | Other and unspecified coagulation defects | 1.52E-02 | 4,484 | 0.044 | 0.018 | 1.52E-02 | -0.015 | 0.011 | 1.88E-01 |
| 318 | Tobacco use disorder | 1.64E-02 | 4,197 | 0.042 | 0.018 | 1.64E-02 | -0.008 | 0.010 | 4.22E-01 |
| 720 | Spinal stenosis | 1.65E-02 | 4,355 | -0.007 | 0.019 | 7.17E-01 | -0.019 | 0.008 | 1.65E-02 |
| 292.3 | Memory loss | 1.74E-02 | 4,299 | -0.045 | 0.019 | 1.74E-02 | -0.011 | 0.006 | 7.77E-02 |
| 274.11 | Gouty arthropathy | 1.87E-02 | 4,460 | 0.013 | 0.022 | 5.47E-01 | -0.026 | 0.011 | 1.87E-02 |
| 480.11 | Pneumococcal pneumonia | 2.04E-02 | 4,490 | 0.024 | 0.010 | 2.04E-02 | 0.000 | 0.005 | 9.27E-01 |
| 271.3 | Intestinal disaccharidase deficiencies and disaccharide malabsorption | 2.07E-02 | 4,306 | 0.009 | 0.010 | 3.54E-01 | -0.010 | 0.004 | 2.07E-02 |
| 596.5 | Functional disorders of bladder | 2.20E-02 | 4,455 | 0.037 | 0.016 | 2.20E-02 | 0.006 | 0.009 | 5.31E-01 |
| 351 | Other peripheral nerve disorders | 2.41E-02 | 4,205 | 0.030 | 0.013 | 2.41E-02 | 0.004 | 0.007 | 5.70E-01 |
| 532 | Dysphagia | 2.55E-02 | 4,240 | 0.026 | 0.012 | 2.55E-02 | 0.001 | 0.006 | 8.15E-01 |
| 289.4 | Lymphadenitis | 2.71E-02 | 4,243 | 0.026 | 0.012 | 2.71E-02 | 0.002 | 0.006 | 7.93E-01 |
| 509.1 | Respiratory failure | 2.79E-02 | 4,460 | 0.035 | 0.016 | 2.79E-02 | -0.006 | 0.008 | 4.52E-01 |
| 537 | Other disorders of stomach and duodenum | 2.81E-02 | 4,326 | -0.013 | 0.015 | 3.86E-01 | -0.013 | 0.006 | 2.81E-02 |
| 411.3 | Angina pectoris | 3.24E-02 | 4,190 | 0.024 | 0.011 | 3.24E-02 | 0.002 | 0.005 | 7.01E-01 |
| 585.1 | Acute renal failure | 3.40E-02 | 4,311 | 0.023 | 0.011 | 3.40E-02 | -0.001 | 0.005 | 9.10E-01 |
| 585.3 | Chronic renal failure [CKD] | 3.98E-02 | 4,250 | 0.026 | 0.012 | 3.98E-02 | 0.002 | 0.006 | 8.02E-01 |
| 458.2 | Iatrogenic hypotension | 4.21E-02 | 4,508 | 0.032 | 0.016 | 4.21E-02 | 0.008 | 0.008 | 3.23E-01 |
| 428.4 | Heart failure with preserved EF [Diastolic heart failure] | 4.43E-02 | 4,399 | 0.035 | 0.018 | 5.00E-02 | 0.018 | 0.009 | 4.43E-02 |
| 428.1 | Congestive heart failure (CHF) NOS | 4.83E-02 | 4,116 | 0.032 | 0.016 | 4.83E-02 | 0.003 | 0.008 | 6.80E-01 |
| 563 | Constipation | 5.01E-02 | 4,079 | 0.018 | 0.009 | 5.01E-02 | -0.006 | 0.004 | 2.05E-01 |
| 290.1 | Dementias | 5.12E-02 | 4,456 | -0.030 | 0.019 | 1.19E-01 | -0.014 | 0.007 | 5.12E-02 |
| 512.9 | Other dyspnea | 5.21E-02 | 3,651 | 0.026 | 0.013 | 5.21E-02 | 0.005 | 0.006 | 4.16E-01 |
| 327.4 | Insomnia | 5.37E-02 | 3,780 | -0.011 | 0.014 | 4.26E-01 | -0.011 | 0.006 | 5.37E-02 |
| 599.9 | Other abnormality of urination | 5.38E-02 | 4,394 | 0.012 | 0.012 | 2.84E-01 | -0.011 | 0.006 | 5.38E-02 |
| 798 | Malaise and fatigue | 5.99E-02 | 3,631 | -0.004 | 0.011 | 7.18E-01 | -0.009 | 0.005 | 5.99E-02 |
| 571.5 | Other chronic nonalcoholic liver disease | 6.06E-02 | 4,357 | 0.025 | 0.013 | 6.06E-02 | 0.001 | 0.007 | 9.00E-01 |
| 578.9 | Hemorrhage of gastrointestinal tract | 6.24E-02 | 4,433 | 0.033 | 0.018 | 6.24E-02 | -0.013 | 0.010 | 1.80E-01 |
| 198.1 | Secondary malignancy of lymph nodes | 6.59E-02 | 4,528 | -0.187 | 0.102 | 6.59E-02 | -0.026 | 0.026 | 3.08E-01 |
| 368 | Visual disturbances | 7.19E-02 | 4,311 | -0.006 | 0.015 | 6.95E-01 | -0.011 | 0.006 | 7.19E-02 |
| 291.8 | Alteration of consciousness | 7.22E-02 | 4,389 | 0.014 | 0.025 | 5.81E-01 | 0.019 | 0.011 | 7.22E-02 |
| 456 | Chronic venous insufficiency [CVI] | 7.94E-02 | 4,441 | 0.015 | 0.022 | 4.92E-01 | -0.019 | 0.011 | 7.94E-02 |
| 427.6 | Premature beats | 8.29E-02 | 4,483 | 0.032 | 0.018 | 8.29E-02 | 0.002 | 0.010 | 8.18E-01 |
| 770 | Myalgia and myositis unspecified | 8.73E-02 | 4,148 | -0.041 | 0.024 | 8.73E-02 | -0.002 | 0.008 | 8.30E-01 |
| 703 | Diseases of nail, NOS | 9.10E-02 | 4,457 | -0.061 | 0.036 | 9.10E-02 | -0.010 | 0.011 | 4.02E-01 |
| 465 | Acute upper respiratory infections of multiple or unspecified sites | 9.14E-02 | 4,040 | 0.019 | 0.011 | 9.14E-02 | 0.001 | 0.005 | 8.48E-01 |
| 599.3 | Dysuria | 9.15E-02 | 4,319 | 0.021 | 0.012 | 9.15E-02 | 0.001 | 0.006 | 8.78E-01 |
| 550.2 | Diaphragmatic hernia | 9.20E-02 | 4,278 | 0.010 | 0.012 | 4.19E-01 | -0.010 | 0.006 | 9.20E-02 |
| 473.4 | Voice disturbance | 9.28E-02 | 4,378 | -0.003 | 0.019 | 8.86E-01 | -0.014 | 0.008 | 9.28E-02 |
| 530.11 | GERD | 9.29E-02 | 3,203 | 0.016 | 0.009 | 9.29E-02 | -0.003 | 0.004 | 4.56E-01 |
| 565.1 | Anal and rectal polyp | 9.36E-02 | 4,377 | 0.016 | 0.010 | 9.36E-02 | -0.002 | 0.005 | 7.35E-01 |
| 599.5 | Frequency of urination and polyuria | 9.40E-02 | 4,207 | -0.016 | 0.016 | 3.05E-01 | -0.010 | 0.006 | 9.40E-02 |
| 280.1 | Iron deficiency anemias, unspecified or not due to blood loss | 9.43E-02 | 4,335 | 0.009 | 0.013 | 5.17E-01 | -0.010 | 0.006 | 9.43E-02 |
| 274.1 | Gout | 1.03E-01 | 4,335 | 0.026 | 0.016 | 1.03E-01 | -0.005 | 0.008 | 5.37E-01 |
| 578.2 | Blood in stool | 1.03E-01 | 4,400 | 0.020 | 0.012 | 1.03E-01 | -0.001 | 0.006 | 8.33E-01 |
| 292.4 | Altered mental status | 1.04E-01 | 4,312 | 0.016 | 0.010 | 1.04E-01 | -0.004 | 0.005 | 3.63E-01 |
| 599.2 | Retention of urine | 1.09E-01 | 4,405 | 0.020 | 0.013 | 1.09E-01 | 0.000 | 0.006 | 9.75E-01 |
| 781 | Symptoms involving nervous and musculoskeletal systems | 1.18E-01 | 4,385 | 0.017 | 0.011 | 1.18E-01 | -0.001 | 0.005 | 7.84E-01 |
| 716.9 | Arthropathy NOS | 1.23E-01 | 4,170 | -0.011 | 0.017 | 5.34E-01 | -0.011 | 0.007 | 1.23E-01 |
| 427.11 | Paroxysmal supraventricular tachycardia | 1.25E-01 | 4,419 | -0.013 | 0.020 | 5.08E-01 | 0.011 | 0.007 | 1.25E-01 |
| 366 | Cataract | 1.29E-01 | 4,359 | -0.009 | 0.019 | 6.33E-01 | -0.012 | 0.008 | 1.29E-01 |
| 512.8 | Cough | 1.32E-01 | 3,600 | 0.015 | 0.010 | 1.32E-01 | 0.006 | 0.004 | 2.03E-01 |
| 472 | Chronic pharyngitis and nasopharyngitis | 1.35E-01 | 4,297 | -0.034 | 0.023 | 1.35E-01 | -0.002 | 0.008 | 7.64E-01 |
| 278.4 | Abnormal weight gain | 1.37E-01 | 4,333 | -0.027 | 0.018 | 1.37E-01 | -0.007 | 0.007 | 3.03E-01 |
| 208 | Benign neoplasm of colon | 1.38E-01 | 3,937 | 0.016 | 0.011 | 1.38E-01 | 0.005 | 0.005 | 3.47E-01 |
| 706.8 | Other specified diseases of sebaceous glands | 1.39E-01 | 4,228 | -0.019 | 0.013 | 1.39E-01 | -0.002 | 0.005 | 6.37E-01 |
| 357 | Inflammatory and toxic neuropathy | 1.39E-01 | 4,342 | 0.016 | 0.011 | 1.39E-01 | 0.002 | 0.005 | 6.88E-01 |
| 687.1 | Rash and other nonspecific skin eruption | 1.49E-01 | 4,108 | 0.012 | 0.009 | 1.69E-01 | -0.006 | 0.004 | 1.49E-01 |
| 681 | Superficial cellulitis and abscess | 1.57E-01 | 4,302 | 0.022 | 0.016 | 1.57E-01 | -0.006 | 0.008 | 4.21E-01 |
| 427.8 | Sinoatrial node dysfunction (Bradycardia) | 1.63E-01 | 4,480 | 0.002 | 0.028 | 9.32E-01 | -0.018 | 0.013 | 1.63E-01 |
| 512.1 | Wheezing | 1.73E-01 | 4,211 | 0.001 | 0.013 | 9.28E-01 | 0.007 | 0.005 | 1.73E-01 |
| 198 | Secondary malignant neoplasm | 1.75E-01 | 4,520 | -0.066 | 0.048 | 1.75E-01 | -0.005 | 0.015 | 7.39E-01 |
| 561.2 | Flatulence | 1.78E-01 | 4,288 | 0.014 | 0.011 | 1.78E-01 | -0.001 | 0.005 | 8.84E-01 |
| 426.31 | Right bundle branch block | 1.84E-01 | 4,463 | 0.017 | 0.016 | 2.78E-01 | 0.010 | 0.007 | 1.84E-01 |
| 386.9 | Dizziness and giddiness (Light-headedness and vertigo) | 1.85E-01 | 4,029 | -0.001 | 0.012 | 9.66E-01 | -0.007 | 0.005 | 1.85E-01 |
| 475 | Chronic sinusitis | 2.09E-01 | 4,240 | -0.020 | 0.016 | 2.09E-01 | -0.003 | 0.006 | 6.06E-01 |
| 276.14 | Hypopotassemia | 2.16E-01 | 4,453 | 0.025 | 0.020 | 2.16E-01 | -0.001 | 0.010 | 9.08E-01 |
| 698 | Pruritus and related conditions | 2.16E-01 | 4,282 | -0.015 | 0.012 | 2.16E-01 | -0.003 | 0.005 | 5.37E-01 |
| 211 | Benign neoplasm of other parts of digestive system | 2.27E-01 | 4,437 | 0.026 | 0.022 | 2.27E-01 | -0.007 | 0.011 | 5.53E-01 |
| 350.2 | Abnormality of gait | 2.32E-01 | 4,340 | -0.022 | 0.021 | 2.82E-01 | -0.009 | 0.008 | 2.32E-01 |
| 278.1 | Obesity | 2.41E-01 | 2,937 | -0.001 | 0.014 | 9.54E-01 | 0.006 | 0.005 | 2.41E-01 |
| 306 | Other mental disorder | 2.47E-01 | 4,428 | -0.029 | 0.025 | 2.47E-01 | 0.001 | 0.009 | 9.24E-01 |
| 726.1 | Enthesopathy | 2.50E-01 | 4,053 | -0.004 | 0.014 | 7.82E-01 | -0.007 | 0.006 | 2.50E-01 |
| 740.9 | Osteoarthrosis NOS | 2.50E-01 | 3,508 | 0.016 | 0.014 | 2.50E-01 | -0.007 | 0.006 | 2.66E-01 |
| 681.5 | Cellulitis and abscess of leg, except foot | 2.57E-01 | 4,427 | 0.017 | 0.015 | 2.57E-01 | 0.003 | 0.007 | 6.97E-01 |
| 250.2 | Type 2 diabetes | 2.65E-01 | 3,738 | -0.007 | 0.021 | 7.38E-01 | 0.009 | 0.008 | 2.65E-01 |
| 427.22 | Atrial flutter | 2.68E-01 | 4,435 | -0.032 | 0.029 | 2.68E-01 | 0.000 | 0.010 | 9.87E-01 |
| 458.1 | Orthostatic hypotension | 2.68E-01 | 4,466 | 0.017 | 0.015 | 2.68E-01 | -0.001 | 0.007 | 8.50E-01 |
| 705.8 | Hyperhidrosis | 2.68E-01 | 4,371 | 0.010 | 0.014 | 4.91E-01 | -0.007 | 0.007 | 2.68E-01 |
| 1019 | Other ill-defined and unknown causes of morbidity and mortality | 2.68E-01 | 4,388 | 0.009 | 0.014 | 5.13E-01 | -0.007 | 0.006 | 2.68E-01 |
| 278 | Overweight, obesity and other hyperalimentation | 2.71E-01 | 3,900 | 0.010 | 0.012 | 4.22E-01 | 0.006 | 0.005 | 2.71E-01 |
| 701 | Other hypertrophic and atrophic conditions of skin | 2.74E-01 | 4,082 | -0.012 | 0.011 | 2.74E-01 | 0.000 | 0.004 | 9.47E-01 |
| 930 | Allergic reaction to food | 2.76E-01 | 4,442 | -0.029 | 0.027 | 2.76E-01 | -0.008 | 0.010 | 3.98E-01 |
| 263 | Other nutritional deficiency | 2.77E-01 | 4,520 | 0.030 | 0.028 | 2.77E-01 | 0.008 | 0.014 | 5.56E-01 |
| 300.4 | Dysthymic disorder | 2.77E-01 | 4,172 | -0.001 | 0.021 | 9.75E-01 | -0.010 | 0.009 | 2.77E-01 |
| 389.1 | Sensorineural hearing loss | 2.91E-01 | 4,363 | -0.026 | 0.024 | 2.91E-01 | -0.001 | 0.009 | 9.33E-01 |
| 202.2 | Non-Hodgkins lymphoma | 2.93E-01 | 4,485 | -0.064 | 0.061 | 2.93E-01 | -0.013 | 0.019 | 4.90E-01 |
| 350.1 | Abnormal involuntary movements | 3.02E-01 | 4,316 | 0.009 | 0.012 | 4.19E-01 | -0.005 | 0.005 | 3.02E-01 |
| 296.22 | Major depressive disorder | 3.14E-01 | 3,465 | 0.006 | 0.008 | 4.62E-01 | -0.003 | 0.003 | 3.14E-01 |
| 338.2 | Chronic pain | 3.20E-01 | 3,973 | 0.009 | 0.009 | 3.20E-01 | -0.002 | 0.004 | 5.98E-01 |
| 481 | Influenza | 3.24E-01 | 4,267 | 0.003 | 0.012 | 8.18E-01 | -0.005 | 0.005 | 3.24E-01 |
| 599.4 | Urinary incontinence | 3.29E-01 | 4,201 | 0.015 | 0.015 | 3.29E-01 | -0.004 | 0.007 | 5.31E-01 |
| 516.1 | Hemoptysis | 3.32E-01 | 4,485 | -0.010 | 0.028 | 7.32E-01 | -0.011 | 0.011 | 3.32E-01 |
| 198.6 | Secondary malignancy of bone | 3.38E-01 | 4,538 | -0.057 | 0.059 | 3.38E-01 | 0.001 | 0.018 | 9.36E-01 |
| 512.7 | Shortness of breath | 3.41E-01 | 3,706 | 0.012 | 0.013 | 3.41E-01 | 0.004 | 0.005 | 4.68E-01 |
| 292.1 | Aphasia/speech disturbance | 3.45E-01 | 4,424 | 0.006 | 0.015 | 6.77E-01 | -0.006 | 0.006 | 3.45E-01 |
| 278.11 | Morbid obesity | 3.52E-01 | 4,005 | -0.021 | 0.023 | 3.52E-01 | 0.001 | 0.008 | 8.73E-01 |
| 427.5 | Arrhythmia (cardiac) NOS | 3.61E-01 | 3,803 | -0.008 | 0.015 | 5.99E-01 | -0.005 | 0.006 | 3.61E-01 |
| 990 | Effects radiation NOS | 3.90E-01 | 4,508 | 0.021 | 0.042 | 6.08E-01 | -0.019 | 0.022 | 3.90E-01 |
| 251.1 | Hypoglycemia | 3.91E-01 | 4,439 | 0.015 | 0.018 | 3.91E-01 | -0.001 | 0.008 | 8.75E-01 |
| 300.12 | Agorophobia, social phobia, and panic disorder | 3.97E-01 | 4,405 | -0.003 | 0.024 | 9.15E-01 | -0.009 | 0.010 | 3.97E-01 |
| 476 | Allergic rhinitis | 4.00E-01 | 3,998 | 0.011 | 0.013 | 4.03E-01 | 0.005 | 0.006 | 4.00E-01 |
| 530.9 | Heartburn | 4.01E-01 | 4,396 | -0.012 | 0.015 | 4.01E-01 | 0.000 | 0.006 | 9.37E-01 |
| 727.1 | Synovitis and tenosynovitis | 4.09E-01 | 4,313 | -0.017 | 0.022 | 4.41E-01 | -0.007 | 0.008 | 4.09E-01 |
| 327 | Sleep disorders | 4.17E-01 | 4,228 | 0.013 | 0.016 | 4.17E-01 | 0.001 | 0.008 | 8.50E-01 |
| 916 | Contusion | 4.17E-01 | 4,182 | 0.009 | 0.011 | 4.17E-01 | -0.002 | 0.005 | 7.47E-01 |
| 327.71 | Restless legs syndrome | 4.29E-01 | 4,321 | -0.018 | 0.023 | 4.29E-01 | -0.007 | 0.009 | 4.66E-01 |
| 433.21 | Cerebral artery occlusion, with cerebral infarction | 4.57E-01 | 4,336 | 0.000 | 0.014 | 9.87E-01 | 0.004 | 0.006 | 4.57E-01 |
| 1005 | Other symptoms | 4.63E-01 | 4,213 | 0.007 | 0.009 | 4.63E-01 | -0.003 | 0.004 | 4.73E-01 |
| 389 | Hearing loss | 4.66E-01 | 4,213 | -0.009 | 0.012 | 4.66E-01 | 0.001 | 0.005 | 8.45E-01 |
| 1002 | Symptoms concerning nutrition, metabolism, and development | 4.66E-01 | 4,358 | 0.010 | 0.013 | 4.72E-01 | -0.004 | 0.006 | 4.66E-01 |
| 272.13 | Mixed hyperlipidemia | 4.74E-01 | 4,221 | -0.004 | 0.013 | 7.78E-01 | -0.004 | 0.005 | 4.74E-01 |
| 495 | Asthma | 4.78E-01 | 3,567 | 0.010 | 0.015 | 4.78E-01 | 0.002 | 0.006 | 8.11E-01 |
| 561 | Symptoms involving digestive system | 4.88E-01 | 4,369 | 0.000 | 0.013 | 9.77E-01 | -0.004 | 0.006 | 4.88E-01 |
| 761 | Cervicalgia | 4.99E-01 | 4,061 | -0.011 | 0.017 | 4.99E-01 | -0.003 | 0.007 | 7.01E-01 |
| 470 | Septal Deviations/Turbinate Hypertrophy | 5.07E-01 | 4,221 | 0.008 | 0.012 | 5.07E-01 | 0.003 | 0.005 | 5.59E-01 |
| 327.1 | Hypersomnia | 5.10E-01 | 3,790 | 0.008 | 0.013 | 5.13E-01 | 0.004 | 0.006 | 5.10E-01 |
| 427.9 | Palpitations | 5.12E-01 | 4,107 | 0.009 | 0.014 | 5.12E-01 | -0.001 | 0.006 | 9.04E-01 |
| 497 | Bronchitis | 5.15E-01 | 4,304 | 0.010 | 0.015 | 5.15E-01 | -0.001 | 0.007 | 9.05E-01 |
| 429.3 | Symptoms involving cardiovascular system | 5.26E-01 | 4,305 | 0.007 | 0.011 | 5.26E-01 | 0.002 | 0.005 | 6.32E-01 |
| 306.9 | Tension headache | 5.39E-01 | 4,293 | -0.010 | 0.017 | 5.39E-01 | 0.002 | 0.007 | 8.02E-01 |
| 415.2 | Chronic pulmonary heart disease | 5.48E-01 | 4,388 | 0.012 | 0.026 | 6.28E-01 | 0.007 | 0.011 | 5.48E-01 |
| 479 | Other upper respiratory disease | 6.05E-01 | 4,285 | 0.002 | 0.014 | 8.64E-01 | 0.003 | 0.006 | 6.05E-01 |
| 915 | Superficial injury without mention of infection | 6.21E-01 | 4,188 | 0.004 | 0.010 | 7.16E-01 | 0.002 | 0.004 | 6.21E-01 |
| 261.4 | Vitamin D deficiency | 6.27E-01 | 4,115 | 0.000 | 0.011 | 9.72E-01 | -0.002 | 0.005 | 6.27E-01 |
| 277 | Other disorders of metabolism | 6.31E-01 | 4,432 | -0.008 | 0.016 | 6.31E-01 | -0.002 | 0.006 | 8.05E-01 |
| 427.21 | Atrial fibrillation | 6.37E-01 | 4,160 | -0.009 | 0.019 | 6.37E-01 | -0.002 | 0.007 | 7.67E-01 |
| 585.4 | Chronic kidney disease, Stage I or II | 6.50E-01 | 4,447 | 0.010 | 0.022 | 6.50E-01 | 0.002 | 0.010 | 8.46E-01 |
| 798.1 | Chronic fatigue syndrome | 6.74E-01 | 4,317 | -0.005 | 0.014 | 7.23E-01 | 0.002 | 0.006 | 6.74E-01 |
| 1009 | Injury, NOS | 6.91E-01 | 3,788 | -0.004 | 0.009 | 6.91E-01 | -0.001 | 0.004 | 7.35E-01 |
| 300.11 | Generalized anxiety disorder | 6.96E-01 | 4,192 | 0.004 | 0.010 | 7.11E-01 | 0.002 | 0.004 | 6.96E-01 |
| 365 | Glaucoma | 7.73E-01 | 4,345 | -0.001 | 0.015 | 9.64E-01 | 0.002 | 0.006 | 7.73E-01 |
| 290.3 | Other persistent mental disorders due to conditions classified elsewhere | 7.99E-01 | 4,461 | 0.021 | 0.084 | 7.99E-01 | 0.000 | 0.041 | 9.93E-01 |
| 427.3 | Other specified cardiac dysrhythmias | 8.34E-01 | 4,242 | 0.003 | 0.013 | 8.34E-01 | 0.001 | 0.006 | 8.64E-01 |
| 313.1 | Attention deficit hyperactivity disorder | 8.47E-01 | 4,365 | 0.004 | 0.023 | 8.47E-01 | 0.001 | 0.010 | 9.46E-01 |
| 279.2 | Autoimmune disease NEC | 1.00E+00 | 4,510 | 0.000 | 516.801 | 1.00E+00 | 0.000 | 217.401 | 1.00E+00 |
| 771.1 | Swelling of limb | 1.00E+00 | 4,314 | 0.000 | 545.454 | 1.00E+00 | 0.000 | 224.813 | 1.00E+00 |
