# Supplementary material for: Sleep apnea phenotyping and relationship to disease in a large clinical biobank
Source: JAMIA Open. 2022 Jan 11;5(1):ooab117. doi: 10.1093/jamiaopen/ooab117 (PMC8826997; doi:10.1093/jamiaopen/ooab117)
Supplement: ooab117_Supplementary_Data [file ooab117_Supplementary_Data.zip › Cade-EHR-sleep-apnea-algorithm-table-s8.doc]

**Table S8. Polysomnograpic Associations with Cross-Sectional PheCode Case Status**

The first PSG recordings from 4,544 participants (with any sleep apnea status) were extracted. All 274 cross-sectional PheCodes that were significant in the combined sex Table S6 analysis were tested.

Multimodal Automated Phenotyping (MAP) was performed similarly to the Table S6 sample, and case/control status was recalibrated based on the cut-offs in the MGB Biobank sample. Logistic regression analyses examined AHI and Per88 (percent of the sleep recording under 88% oxyhemoglobin saturation) and PheCode case status by adjusting for the age and BMI at the time of the PSG, sex, and self-reported population. Clinical encounter count was adjusted for during the MAP phenotype construction. All traits were adjusted for covariates and then rank-normalized. *: Cleared Bonferroni p-value correction.

| **PheCode** | **Translation** | **Min P** | **Per88 Beta** | **Per88 SE** | **Per88 P** | **AHI Beta** | **AHI SE** | **AHI P** |
| --- | --- | --- | --- | --- | --- | --- | --- | --- |
| 1013 | Asphyxia and hypoxemia | 2.10E-41* | 0.065 | 0.005 | 2.10E-41 | 0.002 | 0.003 | 4.04E-01 |
| 496 | Chronic airway obstruction | 4.98E-27* | 0.051 | 0.005 | 4.98E-27 | -0.004 | 0.003 | 9.52E-02 |
| 503 | Pulmonary congestion and hypostasis | 1.90E-22* | 0.051 | 0.005 | 1.90E-22 | 0.014 | 0.003 | 4.46E-07 |
| 415.2 | Chronic pulmonary heart disease | 1.99E-20* | 0.054 | 0.006 | 1.99E-20 | 0.004 | 0.003 | 2.57E-01 |
| 512.7 | Shortness of breath | 3.59E-20* | 0.031 | 0.003 | 3.59E-20 | 0.002 | 0.002 | 2.75E-01 |
| 276.6 | Fluid overload | 3.81E-20* | 0.052 | 0.006 | 3.81E-20 | 0.008 | 0.003 | 5.67E-03 |
| 428.1 | Congestive heart failure (CHF) NOS | 4.31E-20* | 0.038 | 0.004 | 4.31E-20 | 0.007 | 0.002 | 3.88E-04 |
| 509.1 | Respiratory failure | 5.84E-20* | 0.062 | 0.007 | 5.84E-20 | 0.013 | 0.004 | 7.12E-04 |
| 496.1 | Emphysema | 2.61E-18* | 0.056 | 0.006 | 2.61E-18 | -0.004 | 0.004 | 3.47E-01 |
| 782.3 | Edema | 2.06E-16* | 0.032 | 0.004 | 2.06E-16 | 0.002 | 0.002 | 1.91E-01 |
| 508 | Pulmonary collapse; interstitial and compensatory emphysema | 6.50E-16* | 0.038 | 0.005 | 6.50E-16 | 0.002 | 0.002 | 3.32E-01 |
| 512.9 | Other dyspnea | 7.73E-16* | 0.027 | 0.003 | 7.73E-16 | 0.002 | 0.001 | 1.49E-01 |
| 458.9 | Hypotension NOS | 1.82E-14* | 0.044 | 0.006 | 1.82E-14 | 0.007 | 0.003 | 1.35E-02 |
| 428.2 | Heart failure NOS | 5.51E-13* | 0.043 | 0.006 | 5.51E-13 | 0.012 | 0.003 | 2.28E-04 |
| 401.21 | Hypertensive heart disease | 8.26E-13* | 0.042 | 0.006 | 8.26E-13 | 0.008 | 0.003 | 1.39E-02 |
| 428.4 | Heart failure with preserved EF [Diastolic heart failure] | 6.09E-12* | 0.042 | 0.006 | 6.09E-12 | 0.004 | 0.003 | 1.78E-01 |
| 510 | Other diseases of lung | 1.68E-11* | 0.035 | 0.005 | 1.68E-11 | -0.003 | 0.003 | 3.31E-01 |
| 480 | Pneumonia | 1.74E-10* | 0.027 | 0.004 | 1.74E-10 | 0.004 | 0.002 | 3.63E-02 |
| 318 | Tobacco use disorder | 7.81E-10* | 0.028 | 0.005 | 7.81E-10 | 0.001 | 0.002 | 6.87E-01 |
| 505 | Other pulmonary inflamation or edema | 1.34E-09* | 0.048 | 0.008 | 1.34E-09 | 0.013 | 0.004 | 2.03E-03 |
| 428.3 | Heart failure with reduced EF [Systolic or combined heart failure] | 5.22E-09* | 0.045 | 0.008 | 5.22E-09 | 0.010 | 0.004 | 1.78E-02 |
| 585.1 | Acute renal failure | 6.91E-09* | 0.029 | 0.005 | 6.91E-09 | 0.007 | 0.002 | 2.62E-03 |
| 425.1 | Primary/intrinsic cardiomyopathies | 2.53E-08* | 0.033 | 0.006 | 2.53E-08 | 0.014 | 0.003 | 1.26E-06 |
| 316 | Substance addiction and disorders | 8.33E-08* | 0.029 | 0.005 | 8.33E-08 | 0.003 | 0.003 | 1.85E-01 |
| 411.2 | Myocardial infarction | 9.88E-08* | 0.027 | 0.005 | 9.88E-08 | 0.007 | 0.002 | 3.13E-03 |
| 507 | Pleurisy; pleural effusion | 1.20E-07* | 0.029 | 0.005 | 1.20E-07 | 0.006 | 0.003 | 2.25E-02 |
| 276.13 | Hyperpotassemia | 8.12E-07* | 0.038 | 0.008 | 8.12E-07 | 0.016 | 0.004 | 7.03E-05 |
| 416 | Cardiomegaly | 1.19E-06* | 0.032 | 0.007 | 1.19E-06 | 0.012 | 0.003 | 3.55E-04 |
| 427.7 | Tachycardia NOS | 1.79E-06* | 0.023 | 0.005 | 1.79E-06 | 0.005 | 0.002 | 3.79E-02 |
| 250.2 | Type 2 diabetes | 2.03E-06* | 0.017 | 0.004 | 2.03E-06 | 0.005 | 0.002 | 1.16E-03 |
| 285 | Other anemias | 2.34E-06* | 0.017 | 0.004 | 2.34E-06 | 0.003 | 0.002 | 9.85E-02 |
| 539 | Bariatric surgery | 3.47E-06* | -0.023 | 0.006 | 3.36E-05 | -0.010 | 0.002 | 3.47E-06 |
| 038 | Septicemia | 6.06E-06* | 0.034 | 0.008 | 6.06E-06 | 0.008 | 0.004 | 4.91E-02 |
| 327.4 | Insomnia | 6.25E-06* | 0.000 | 0.004 | 9.72E-01 | -0.007 | 0.002 | 6.25E-06 |
| 260 | Protein-calorie malnutrition | 1.11E-05* | 0.034 | 0.008 | 1.11E-05 | 0.006 | 0.004 | 1.27E-01 |
| 401.1 | Essential hypertension | 1.14E-05* | 0.008 | 0.003 | 9.31E-03 | 0.005 | 0.001 | 1.14E-05 |
| 340 | Migraine | 1.62E-05* | -0.015 | 0.004 | 1.14E-03 | -0.008 | 0.002 | 1.62E-05 |
| 512.1 | Wheezing | 1.68E-05* | 0.020 | 0.005 | 1.68E-05 | -0.003 | 0.002 | 2.08E-01 |
| 458.2 | Iatrogenic hypotension | 4.33E-05* | 0.040 | 0.010 | 4.33E-05 | 0.009 | 0.005 | 9.22E-02 |
| 427.5 | Arrhythmia (cardiac) NOS | 4.84E-05* | 0.015 | 0.004 | 4.84E-05 | 0.002 | 0.002 | 2.82E-01 |
| 411.3 | Angina pectoris | 5.96E-05* | 0.018 | 0.005 | 5.96E-05 | 0.002 | 0.002 | 2.49E-01 |
| 427.22 | Atrial flutter | 1.22E-04 | 0.028 | 0.007 | 1.22E-04 | 0.006 | 0.004 | 1.26E-01 |
| 276.41 | Acidosis | 1.40E-04 | 0.029 | 0.008 | 1.40E-04 | 0.011 | 0.004 | 2.90E-03 |
| 578.9 | Hemorrhage of gastrointestinal tract | 2.52E-04 | 0.027 | 0.007 | 2.52E-04 | 0.004 | 0.004 | 2.87E-01 |
| 771.1 | Swelling of limb | 3.10E-04 | 0.021 | 0.006 | 3.10E-04 | 0.000 | 0.003 | 9.79E-01 |
| 585.3 | Chronic renal failure [CKD] | 3.15E-04 | 0.018 | 0.005 | 3.15E-04 | 0.006 | 0.002 | 1.54E-02 |
| 501 | Pneumonitis due to inhalation of food or vomitus | 3.34E-04 | 0.042 | 0.012 | 3.34E-04 | 0.005 | 0.007 | 4.51E-01 |
| 979 | Adverse drug events and drug allergies | 3.88E-04 | 0.018 | 0.005 | 3.88E-04 | 0.001 | 0.002 | 7.88E-01 |
| 1015 | Effects of other external causes | 7.72E-04 | 0.016 | 0.005 | 7.72E-04 | 0.002 | 0.002 | 4.25E-01 |
| 429.3 | Symptoms involving cardiovascular system | 8.50E-04 | 0.017 | 0.005 | 8.50E-04 | 0.004 | 0.002 | 7.13E-02 |
| 764 | Sciatica | 1.05E-03 | 0.016 | 0.005 | 1.05E-03 | 0.002 | 0.002 | 2.93E-01 |
| 729 | Other disorders of soft tissues | 1.15E-03 | 0.016 | 0.005 | 1.15E-03 | 0.000 | 0.002 | 8.39E-01 |
| 276.5 | Hypovolemia | 1.19E-03 | 0.016 | 0.005 | 1.19E-03 | 0.001 | 0.002 | 5.83E-01 |
| 512.8 | Cough | 1.21E-03 | 0.011 | 0.003 | 1.21E-03 | 0.002 | 0.001 | 1.18E-01 |
| 427.21 | Atrial fibrillation | 1.40E-03 | 0.015 | 0.005 | 1.40E-03 | 0.001 | 0.002 | 6.21E-01 |
| 797 | Shock | 1.56E-03 | 0.037 | 0.012 | 1.56E-03 | 0.001 | 0.006 | 8.72E-01 |
| 477 | Epistaxis or throat hemorrhage | 1.62E-03 | 0.023 | 0.007 | 1.62E-03 | 0.003 | 0.004 | 3.77E-01 |
| 041 | Bacterial infection NOS | 1.65E-03 | 0.020 | 0.009 | 2.21E-02 | 0.013 | 0.004 | 1.65E-03 |
| 300.9 | Posttraumatic stress disorder | 1.74E-03 | 0.007 | 0.006 | 3.09E-01 | -0.009 | 0.003 | 1.74E-03 |
| 789 | Nausea and vomiting | 1.90E-03 | 0.001 | 0.004 | 8.36E-01 | -0.006 | 0.002 | 1.90E-03 |
| 414 | Other forms of chronic heart disease | 2.27E-03 | 0.023 | 0.008 | 2.27E-03 | 0.009 | 0.004 | 8.37E-03 |
| 411.4 | Coronary atherosclerosis | 2.29E-03 | 0.014 | 0.005 | 2.29E-03 | 0.004 | 0.002 | 2.92E-02 |
| 459.9 | Circulatory disease NEC | 2.38E-03 | 0.016 | 0.005 | 2.38E-03 | 0.000 | 0.002 | 8.39E-01 |
| 721.1 | Spondylosis without myelopathy | 2.56E-03 | -0.008 | 0.005 | 1.42E-01 | -0.007 | 0.002 | 2.56E-03 |
| 292.4 | Altered mental status | 2.57E-03 | 0.016 | 0.005 | 2.57E-03 | 0.000 | 0.002 | 9.41E-01 |
| 687.1 | Rash and other nonspecific skin eruption | 2.72E-03 | -0.007 | 0.005 | 1.36E-01 | -0.006 | 0.002 | 2.72E-03 |
| 585.2 | Renal failure NOS | 2.75E-03 | 0.026 | 0.009 | 2.75E-03 | 0.011 | 0.004 | 6.32E-03 |
| 418 | Nonspecific chest pain | 2.76E-03 | 0.010 | 0.003 | 2.76E-03 | 0.003 | 0.001 | 1.91E-02 |
| 681 | Superficial cellulitis and abscess | 2.76E-03 | 0.017 | 0.006 | 2.76E-03 | -0.001 | 0.003 | 7.66E-01 |
| 339 | Other headache syndromes | 3.25E-03 | -0.008 | 0.004 | 4.70E-02 | -0.005 | 0.002 | 3.25E-03 |
| 250.42 | Other abnormal glucose | 3.60E-03 | 0.013 | 0.005 | 3.60E-03 | 0.005 | 0.002 | 7.47E-03 |
| 286.7 | Other and unspecified coagulation defects | 3.68E-03 | 0.027 | 0.009 | 3.68E-03 | 0.001 | 0.005 | 8.77E-01 |
| 395.1 | Nonrheumatic mitral valve disorders | 3.78E-03 | 0.019 | 0.007 | 3.78E-03 | -0.001 | 0.003 | 8.02E-01 |
| 269 | Proteinuria | 3.81E-03 | 0.007 | 0.007 | 3.15E-01 | 0.008 | 0.003 | 3.81E-03 |
| 743.11 | Osteoporosis NOS | 4.15E-03 | 0.014 | 0.005 | 4.15E-03 | 0.003 | 0.002 | 1.72E-01 |
| 395.2 | Nonrheumatic aortic valve disorders | 4.52E-03 | 0.021 | 0.007 | 4.52E-03 | 0.005 | 0.003 | 1.56E-01 |
| 459.1 | Hemorrhage NOS | 4.76E-03 | 0.025 | 0.009 | 4.76E-03 | -0.003 | 0.005 | 4.43E-01 |
| 401.3 | Other hypertensive complications | 4.79E-03 | 0.012 | 0.009 | 1.96E-01 | 0.011 | 0.004 | 4.79E-03 |
| 250.1 | Type 1 diabetes | 4.84E-03 | 0.018 | 0.008 | 2.36E-02 | 0.010 | 0.004 | 4.84E-03 |
| 401.22 | Hypertensive chronic kidney disease | 5.19E-03 | 0.015 | 0.006 | 1.57E-02 | 0.008 | 0.003 | 5.19E-03 |
| 578.2 | Blood in stool | 5.50E-03 | 0.018 | 0.006 | 5.50E-03 | 0.000 | 0.003 | 9.87E-01 |
| 251.1 | Hypoglycemia | 5.78E-03 | 0.017 | 0.008 | 2.73E-02 | 0.010 | 0.004 | 5.78E-03 |
| 288.2 | Elevated white blood cell count | 6.30E-03 | 0.015 | 0.006 | 6.30E-03 | 0.002 | 0.003 | 4.47E-01 |
| 1009 | Injury, NOS | 6.89E-03 | 0.010 | 0.004 | 6.89E-03 | -0.001 | 0.002 | 5.64E-01 |
| 368.2 | Diplopia and disorders of binocular vision | 6.96E-03 | -0.012 | 0.012 | 3.00E-01 | -0.013 | 0.005 | 6.96E-03 |
| 564 | Functional digestive disorders | 8.39E-03 | 0.011 | 0.009 | 2.06E-01 | -0.011 | 0.004 | 8.39E-03 |
| 519.8 | Other diseases of respiratory system, NEC | 8.70E-03 | 0.019 | 0.007 | 8.70E-03 | 0.004 | 0.003 | 2.84E-01 |
| 530.14 | Reflux esophagitis | 9.10E-03 | -0.021 | 0.008 | 9.10E-03 | -0.004 | 0.003 | 2.20E-01 |
| 495 | Asthma | 9.98E-03 | 0.000 | 0.003 | 9.10E-01 | -0.004 | 0.001 | 9.98E-03 |
| 586 | Other disorders of the kidney and ureters | 1.02E-02 | 0.014 | 0.006 | 1.02E-02 | 0.004 | 0.003 | 1.25E-01 |
| 276.14 | Hypopotassemia | 1.03E-02 | 0.021 | 0.008 | 1.03E-02 | 0.003 | 0.004 | 3.80E-01 |
| 296.22 | Major depressive disorder | 1.03E-02 | -0.001 | 0.003 | 6.98E-01 | -0.004 | 0.001 | 1.03E-02 |
| 348.8 | Encephalopathy, not elsewhere classified | 1.03E-02 | 0.023 | 0.009 | 1.03E-02 | 0.008 | 0.004 | 5.17E-02 |
| 504 | Other alveolar and parietoalveolar pneumonopathy | 1.04E-02 | 0.025 | 0.010 | 1.04E-02 | -0.006 | 0.005 | 2.60E-01 |
| 728.7 | Fasciitis | 1.09E-02 | -0.017 | 0.007 | 1.09E-02 | -0.007 | 0.003 | 1.38E-02 |
| 280.2 | Iron deficiency anemia secondary to blood loss (chronic) | 1.09E-02 | 0.022 | 0.009 | 1.09E-02 | -0.006 | 0.004 | 1.99E-01 |
| 278.4 | Abnormal weight gain | 1.26E-02 | -0.013 | 0.007 | 6.55E-02 | -0.007 | 0.003 | 1.26E-02 |
| 793.2 | Nonspecific abnormal findings on radiological and other examination of other intrathoracic organs (echocardiogram, etc) | 1.27E-02 | 0.016 | 0.006 | 1.27E-02 | -0.003 | 0.003 | 3.93E-01 |
| 285.1 | Acute posthemorrhagic anemia | 1.51E-02 | 0.021 | 0.010 | 2.90E-02 | 0.011 | 0.005 | 1.51E-02 |
| 532 | Dysphagia | 1.60E-02 | 0.012 | 0.005 | 1.60E-02 | -0.001 | 0.002 | 5.24E-01 |
| 760 | Back pain | 1.61E-02 | -0.002 | 0.003 | 4.74E-01 | -0.003 | 0.001 | 1.61E-02 |
| 747.11 | Cardiac shunt/ heart septal defect | 1.69E-02 | 0.019 | 0.008 | 1.69E-02 | 0.006 | 0.004 | 9.95E-02 |
| 480.11 | Pneumococcal pneumonia | 1.73E-02 | 0.019 | 0.008 | 1.73E-02 | 0.004 | 0.004 | 3.09E-01 |
| 345 | Epilepsy, recurrent seizures, convulsions | 1.76E-02 | -0.002 | 0.007 | 7.64E-01 | -0.007 | 0.003 | 1.76E-02 |
| 681.5 | Cellulitis and abscess of leg, except foot | 1.92E-02 | 0.017 | 0.007 | 1.92E-02 | 0.001 | 0.003 | 6.74E-01 |
| 327.1 | Hypersomnia | 1.99E-02 | 0.009 | 0.004 | 1.99E-02 | 0.000 | 0.002 | 9.70E-01 |
| 356 | Hereditary and idiopathic peripheral neuropathy | 2.11E-02 | 0.013 | 0.006 | 2.11E-02 | 0.000 | 0.003 | 9.28E-01 |
| 530.9 | Heartburn | 2.21E-02 | -0.018 | 0.008 | 2.21E-02 | -0.006 | 0.003 | 6.32E-02 |
| 698 | Pruritus and related conditions | 2.26E-02 | -0.014 | 0.006 | 2.26E-02 | -0.002 | 0.002 | 4.34E-01 |
| 338.2 | Chronic pain | 2.30E-02 | 0.009 | 0.004 | 2.30E-02 | 0.000 | 0.002 | 7.72E-01 |
| 292.3 | Memory loss | 2.39E-02 | -0.015 | 0.006 | 2.39E-02 | -0.003 | 0.003 | 2.42E-01 |
| 563 | Constipation | 2.45E-02 | 0.010 | 0.004 | 2.45E-02 | -0.002 | 0.002 | 2.53E-01 |
| 290.2 | Delirium due to conditions classified elsewhere | 2.73E-02 | 0.020 | 0.009 | 2.73E-02 | -0.003 | 0.004 | 5.26E-01 |
| 426.92 | Cardiac defibrillator in situ | 2.79E-02 | 0.021 | 0.010 | 4.22E-02 | 0.011 | 0.005 | 2.79E-02 |
| 497 | Bronchitis | 2.93E-02 | 0.012 | 0.006 | 2.93E-02 | -0.001 | 0.003 | 8.07E-01 |
| 300.4 | Dysthymic disorder | 2.94E-02 | -0.008 | 0.005 | 1.40E-01 | -0.005 | 0.002 | 2.94E-02 |
| 274.11 | Gouty arthropathy | 3.03E-02 | 0.019 | 0.009 | 3.03E-02 | 0.005 | 0.004 | 2.40E-01 |
| 591 | Urinary tract infection | 3.08E-02 | 0.010 | 0.005 | 3.08E-02 | 0.001 | 0.002 | 6.15E-01 |
| 427.8 | Sinoatrial node dysfunction (Bradycardia) | 3.50E-02 | 0.021 | 0.010 | 3.50E-02 | -0.005 | 0.005 | 3.10E-01 |
| 357 | Inflammatory and toxic neuropathy | 3.62E-02 | 0.012 | 0.006 | 3.62E-02 | 0.003 | 0.003 | 3.06E-01 |
| 452.2 | Deep vein thrombosis [DVT] | 3.66E-02 | 0.014 | 0.007 | 3.66E-02 | -0.004 | 0.003 | 2.24E-01 |
| 599.9 | Other abnormality of urination | 3.71E-02 | -0.002 | 0.007 | 8.20E-01 | -0.006 | 0.003 | 3.71E-02 |
| 272.1 | Hyperlipidemia | 3.72E-02 | 0.002 | 0.003 | 4.47E-01 | 0.003 | 0.001 | 3.72E-02 |
| 368 | Visual disturbances | 3.93E-02 | -0.004 | 0.006 | 4.86E-01 | -0.005 | 0.003 | 3.93E-02 |
| 427.9 | Palpitations | 3.94E-02 | 0.000 | 0.005 | 9.82E-01 | -0.004 | 0.002 | 3.94E-02 |
| 788 | Syncope and collapse | 4.08E-02 | 0.010 | 0.005 | 4.08E-02 | -0.001 | 0.002 | 6.96E-01 |
| 426.21 | First degree AV block | 4.08E-02 | 0.019 | 0.010 | 4.08E-02 | 0.008 | 0.004 | 5.88E-02 |
| 798 | Malaise and fatigue | 4.15E-02 | 0.007 | 0.003 | 4.15E-02 | 0.000 | 0.001 | 8.57E-01 |
| 770 | Myalgia and myositis unspecified | 4.24E-02 | -0.002 | 0.005 | 7.43E-01 | -0.004 | 0.002 | 4.24E-02 |
| 949 | Allergies, other | 4.27E-02 | 0.003 | 0.005 | 5.44E-01 | -0.005 | 0.002 | 4.27E-02 |
| 348.7 | Coma | 4.90E-02 | 0.019 | 0.010 | 4.90E-02 | -0.001 | 0.005 | 8.74E-01 |
| 350.1 | Abnormal involuntary movements | 4.93E-02 | 0.011 | 0.006 | 4.93E-02 | -0.003 | 0.003 | 1.98E-01 |
| 291.8 | Alteration of consciousness | 4.96E-02 | 0.014 | 0.007 | 4.96E-02 | 0.005 | 0.003 | 1.30E-01 |
| 476 | Allergic rhinitis | 5.05E-02 | -0.009 | 0.004 | 5.05E-02 | -0.002 | 0.002 | 3.31E-01 |
| 561 | Symptoms involving digestive system | 5.15E-02 | -0.003 | 0.007 | 6.90E-01 | -0.006 | 0.003 | 5.15E-02 |
| 456 | Chronic venous insufficiency [CVI] | 5.25E-02 | 0.016 | 0.008 | 5.25E-02 | -0.004 | 0.004 | 3.10E-01 |
| 455 | Hemorrhoids | 5.58E-02 | -0.005 | 0.004 | 2.22E-01 | -0.003 | 0.002 | 5.58E-02 |
| 396 | Abnormal heart sounds | 5.68E-02 | 0.005 | 0.006 | 4.04E-01 | 0.005 | 0.003 | 5.68E-02 |
| 327 | Sleep disorders | 5.84E-02 | -0.006 | 0.006 | 3.03E-01 | -0.004 | 0.002 | 5.84E-02 |
| 550.4 | Umbilical hernia | 6.10E-02 | 0.016 | 0.009 | 6.10E-02 | 0.005 | 0.004 | 2.43E-01 |
| 458.1 | Orthostatic hypotension | 6.40E-02 | 0.016 | 0.008 | 6.40E-02 | 0.000 | 0.004 | 9.73E-01 |
| 394.7 | Disease of tricuspid valve | 7.06E-02 | 0.024 | 0.013 | 7.06E-02 | 0.001 | 0.007 | 8.59E-01 |
| 781 | Symptoms involving nervous and musculoskeletal systems | 7.08E-02 | 0.011 | 0.006 | 7.08E-02 | -0.001 | 0.003 | 6.51E-01 |
| 274.1 | Gout | 7.24E-02 | 0.010 | 0.006 | 1.07E-01 | 0.005 | 0.003 | 7.24E-02 |
| 726.1 | Enthesopathy | 7.56E-02 | -0.008 | 0.005 | 7.56E-02 | -0.002 | 0.002 | 3.43E-01 |
| 252.1 | Hyperparathyroidism | 7.57E-02 | -0.020 | 0.011 | 7.57E-02 | 0.000 | 0.004 | 9.96E-01 |
| 280.1 | Iron deficiency anemias, unspecified or not due to blood loss | 7.77E-02 | 0.006 | 0.006 | 3.28E-01 | -0.005 | 0.003 | 7.77E-02 |
| 345.3 | Convulsions | 7.89E-02 | 0.002 | 0.006 | 7.55E-01 | -0.005 | 0.003 | 7.89E-02 |
| 701 | Other hypertrophic and atrophic conditions of skin | 9.06E-02 | -0.008 | 0.005 | 9.06E-02 | 0.000 | 0.002 | 9.43E-01 |
| 250.41 | Impaired fasting glucose | 9.64E-02 | 0.009 | 0.005 | 9.64E-02 | 0.003 | 0.002 | 2.26E-01 |
| 530.2 | Esophageal bleeding (varices/hemorrhage) | 1.01E-01 | 0.016 | 0.010 | 1.01E-01 | -0.001 | 0.005 | 8.64E-01 |
| 296.1 | Bipolar | 1.02E-01 | 0.010 | 0.006 | 1.02E-01 | 0.003 | 0.003 | 3.63E-01 |
| 916 | Contusion | 1.04E-01 | 0.008 | 0.005 | 1.04E-01 | 0.000 | 0.002 | 9.15E-01 |
| 537 | Other disorders of stomach and duodenum | 1.05E-01 | 0.002 | 0.006 | 7.63E-01 | -0.004 | 0.003 | 1.05E-01 |
| 565.1 | Anal and rectal polyp | 1.05E-01 | 0.010 | 0.006 | 1.05E-01 | 0.001 | 0.003 | 7.11E-01 |
| 433.21 | Cerebral artery occlusion, with cerebral infarction | 1.05E-01 | -0.001 | 0.006 | 9.34E-01 | 0.004 | 0.003 | 1.05E-01 |
| 427.12 | Paroxysmal ventricular tachycardia | 1.06E-01 | 0.015 | 0.009 | 1.06E-01 | 0.004 | 0.004 | 4.23E-01 |
| 727 | Other disorders of synovium, tendon, and bursa | 1.08E-01 | -0.018 | 0.011 | 1.08E-01 | -0.006 | 0.004 | 1.68E-01 |
| 703 | Diseases of nail, NOS | 1.08E-01 | 0.014 | 0.009 | 1.08E-01 | 0.002 | 0.004 | 5.63E-01 |
| 198.1 | Secondary malignancy of lymph nodes | 1.12E-01 | -0.046 | 0.029 | 1.12E-01 | 0.006 | 0.009 | 5.30E-01 |
| 277 | Other disorders of metabolism | 1.15E-01 | -0.013 | 0.009 | 1.35E-01 | -0.005 | 0.003 | 1.15E-01 |
| 593 | Hematuria | 1.18E-01 | -0.003 | 0.006 | 6.47E-01 | 0.004 | 0.002 | 1.18E-01 |
| 550.2 | Diaphragmatic hernia | 1.21E-01 | -0.006 | 0.006 | 3.25E-01 | -0.004 | 0.002 | 1.21E-01 |
| 427.11 | Paroxysmal supraventricular tachycardia | 1.26E-01 | 0.011 | 0.007 | 1.26E-01 | 0.003 | 0.003 | 3.02E-01 |
| 452 | Other venous embolism and thrombosis | 1.32E-01 | 0.009 | 0.006 | 1.54E-01 | -0.004 | 0.003 | 1.32E-01 |
| 276.1 | Electrolyte imbalance | 1.35E-01 | 0.020 | 0.013 | 1.35E-01 | -0.005 | 0.007 | 4.40E-01 |
| 773 | Pain in limb | 1.38E-01 | -0.001 | 0.003 | 7.67E-01 | -0.002 | 0.001 | 1.38E-01 |
| 599.2 | Retention of urine | 1.40E-01 | 0.010 | 0.007 | 1.40E-01 | 0.002 | 0.003 | 4.58E-01 |
| 516.1 | Hemoptysis | 1.41E-01 | 0.015 | 0.010 | 1.41E-01 | 0.002 | 0.005 | 6.48E-01 |
| 300.1 | Anxiety disorder | 1.41E-01 | 0.002 | 0.003 | 6.44E-01 | -0.002 | 0.001 | 1.41E-01 |
| 530.11 | GERD | 1.43E-01 | 0.005 | 0.003 | 1.43E-01 | 0.000 | 0.001 | 8.58E-01 |
| 365 | Glaucoma | 1.43E-01 | 0.005 | 0.006 | 4.12E-01 | 0.004 | 0.003 | 1.43E-01 |
| 479 | Other upper respiratory disease | 1.47E-01 | 0.008 | 0.006 | 1.47E-01 | 0.003 | 0.002 | 2.30E-01 |
| 278.1 | Obesity | 1.50E-01 | -0.004 | 0.003 | 1.50E-01 | 0.001 | 0.001 | 3.91E-01 |
| 481 | Influenza | 1.54E-01 | 0.007 | 0.005 | 1.54E-01 | -0.002 | 0.002 | 3.82E-01 |
| 386.9 | Dizziness and giddiness (Light-headedness and vertigo) | 1.56E-01 | -0.002 | 0.004 | 6.29E-01 | -0.003 | 0.002 | 1.56E-01 |
| 389 | Hearing loss | 1.56E-01 | -0.007 | 0.005 | 1.56E-01 | 0.000 | 0.002 | 8.24E-01 |
| 706.8 | Other specified diseases of sebaceous glands | 1.58E-01 | -0.008 | 0.005 | 1.58E-01 | -0.002 | 0.002 | 3.69E-01 |
| 465.2 | Acute pharyngitis | 1.65E-01 | -0.008 | 0.006 | 1.65E-01 | -0.001 | 0.002 | 7.97E-01 |
| 261.2 | Vitamin B-complex deficiencies | 1.67E-01 | -0.014 | 0.010 | 1.67E-01 | -0.001 | 0.004 | 8.34E-01 |
| 263 | Other nutritional deficiency | 1.69E-01 | 0.021 | 0.015 | 1.69E-01 | -0.002 | 0.007 | 8.28E-01 |
| 411.1 | Unstable angina (intermediate coronary syndrome) | 1.70E-01 | 0.009 | 0.008 | 2.26E-01 | 0.005 | 0.003 | 1.70E-01 |
| 261.4 | Vitamin D deficiency | 1.71E-01 | -0.007 | 0.005 | 1.71E-01 | -0.001 | 0.002 | 5.81E-01 |
| 475.9 | Postnasal drip | 1.72E-01 | 0.007 | 0.007 | 3.61E-01 | -0.004 | 0.003 | 1.72E-01 |
| 290.3 | Other persistent mental disorders due to conditions classified elsewhere | 1.73E-01 | -0.005 | 0.011 | 6.29E-01 | 0.006 | 0.004 | 1.73E-01 |
| 715 | Other inflammatory spondylopathies | 1.81E-01 | -0.008 | 0.011 | 4.90E-01 | -0.006 | 0.005 | 1.81E-01 |
| 427.3 | Other specified cardiac dysrhythmias | 1.83E-01 | 0.007 | 0.005 | 1.83E-01 | 0.000 | 0.002 | 9.23E-01 |
| 695.9 | Unspecified erythematous condition | 1.86E-01 | 0.007 | 0.005 | 1.86E-01 | 0.003 | 0.002 | 2.46E-01 |
| 208 | Benign neoplasm of colon | 1.89E-01 | 0.005 | 0.004 | 1.89E-01 | 0.000 | 0.002 | 8.77E-01 |
| 772.2 | Spasm of muscle | 1.89E-01 | -0.010 | 0.008 | 2.32E-01 | -0.004 | 0.003 | 1.89E-01 |
| 389.1 | Sensorineural hearing loss | 1.95E-01 | -0.006 | 0.007 | 4.35E-01 | -0.004 | 0.003 | 1.95E-01 |
| 418.1 | Precordial pain | 1.99E-01 | 0.010 | 0.008 | 1.99E-01 | -0.003 | 0.003 | 4.23E-01 |
| 687.4 | Disturbance of skin sensation | 2.02E-01 | -0.002 | 0.005 | 6.32E-01 | -0.003 | 0.002 | 2.02E-01 |
| 557 | Intestinal malabsorption (non-celiac) | 2.05E-01 | 0.002 | 0.010 | 8.51E-01 | -0.006 | 0.005 | 2.05E-01 |
| 427.6 | Premature beats | 2.07E-01 | 0.013 | 0.010 | 2.07E-01 | -0.002 | 0.005 | 7.32E-01 |
| 351 | Other peripheral nerve disorders | 2.09E-01 | 0.006 | 0.005 | 2.09E-01 | -0.002 | 0.002 | 4.61E-01 |
| 272.11 | Hypercholesterolemia | 2.10E-01 | -0.005 | 0.004 | 2.10E-01 | 0.002 | 0.002 | 3.07E-01 |
| 278.11 | Morbid obesity | 2.11E-01 | 0.005 | 0.004 | 2.11E-01 | -0.002 | 0.002 | 3.16E-01 |
| 599.8 | Other symptoms involving urinary system | 2.13E-01 | 0.012 | 0.010 | 2.13E-01 | -0.004 | 0.005 | 3.94E-01 |
| 766 | Neuralgia, neuritis, and radiculitis NOS | 2.20E-01 | 0.009 | 0.007 | 2.20E-01 | -0.002 | 0.003 | 6.01E-01 |
| 244.4 | Hypothyroidism NOS | 2.24E-01 | 0.005 | 0.004 | 2.24E-01 | -0.002 | 0.002 | 3.51E-01 |
| 930 | Allergic reaction to food | 2.30E-01 | 0.010 | 0.008 | 2.30E-01 | 0.002 | 0.004 | 5.67E-01 |
| 798.1 | Chronic fatigue syndrome | 2.35E-01 | -0.007 | 0.006 | 2.96E-01 | -0.003 | 0.003 | 2.35E-01 |
| 470 | Septal Deviations/Turbinate Hypertrophy | 2.41E-01 | -0.006 | 0.005 | 2.41E-01 | 0.000 | 0.002 | 9.34E-01 |
| 695.8 | Other specified erythematous conditions | 2.43E-01 | -0.015 | 0.013 | 2.54E-01 | -0.006 | 0.005 | 2.43E-01 |
| 599.3 | Dysuria | 2.46E-01 | 0.007 | 0.006 | 2.46E-01 | 0.001 | 0.003 | 6.97E-01 |
| 198 | Secondary malignant neoplasm | 2.47E-01 | -0.023 | 0.020 | 2.47E-01 | -0.001 | 0.007 | 8.40E-01 |
| 740.9 | Osteoarthrosis NOS | 2.47E-01 | 0.004 | 0.003 | 2.47E-01 | -0.002 | 0.001 | 2.62E-01 |
| 327.71 | Restless legs syndrome | 2.62E-01 | -0.003 | 0.007 | 6.61E-01 | -0.003 | 0.003 | 2.62E-01 |
| 306 | Other mental disorder | 2.65E-01 | -0.010 | 0.009 | 2.65E-01 | -0.002 | 0.004 | 5.05E-01 |
| 112 | Candidiasis | 2.68E-01 | 0.006 | 0.006 | 2.68E-01 | 0.001 | 0.002 | 5.34E-01 |
| 783 | Fever of unknown origin | 2.69E-01 | 0.005 | 0.005 | 2.69E-01 | 0.001 | 0.002 | 4.82E-01 |
| 785 | Abdominal pain | 2.69E-01 | -0.001 | 0.003 | 6.96E-01 | -0.002 | 0.001 | 2.69E-01 |
| 338.1 | Acute pain | 2.75E-01 | 0.003 | 0.004 | 5.17E-01 | -0.002 | 0.002 | 2.75E-01 |
| 585.4 | Chronic kidney disease, Stage I or II | 2.94E-01 | -0.001 | 0.009 | 8.93E-01 | 0.004 | 0.004 | 2.94E-01 |
| 564.1 | Irritable Bowel Syndrome | 3.14E-01 | -0.007 | 0.007 | 3.14E-01 | -0.002 | 0.003 | 4.58E-01 |
| 915 | Superficial injury without mention of infection | 3.18E-01 | -0.002 | 0.005 | 6.13E-01 | -0.002 | 0.002 | 3.18E-01 |
| 008.6 | Viral Enteritis | 3.18E-01 | -0.009 | 0.009 | 3.18E-01 | -0.002 | 0.004 | 4.88E-01 |
| 304 | Adjustment reaction | 3.38E-01 | 0.005 | 0.005 | 3.38E-01 | -0.001 | 0.002 | 7.84E-01 |
| 313.1 | Attention deficit hyperactivity disorder | 3.43E-01 | -0.007 | 0.007 | 3.43E-01 | -0.002 | 0.003 | 5.35E-01 |
| 381.11 | Suppurative and unspecified otitis media | 3.52E-01 | 0.007 | 0.008 | 3.52E-01 | 0.000 | 0.003 | 9.40E-01 |
| 716.9 | Arthropathy NOS | 3.67E-01 | 0.003 | 0.005 | 5.38E-01 | -0.002 | 0.002 | 3.67E-01 |
| 279.2 | Autoimmune disease NEC | 3.72E-01 | 0.014 | 0.015 | 3.72E-01 | -0.004 | 0.007 | 5.92E-01 |
| 761 | Cervicalgia | 3.74E-01 | -0.002 | 0.005 | 7.39E-01 | -0.002 | 0.002 | 3.74E-01 |
| 433.8 | Late effects of cerebrovascular disease | 3.75E-01 | 0.005 | 0.009 | 5.76E-01 | 0.004 | 0.004 | 3.75E-01 |
| 300.11 | Generalized anxiety disorder | 3.80E-01 | -0.002 | 0.005 | 7.07E-01 | -0.002 | 0.002 | 3.80E-01 |
| 599.5 | Frequency of urination and polyuria | 3.81E-01 | -0.003 | 0.005 | 5.62E-01 | -0.002 | 0.002 | 3.81E-01 |
| 561.2 | Flatulence | 3.86E-01 | 0.005 | 0.005 | 3.86E-01 | 0.001 | 0.002 | 5.51E-01 |
| 571.5 | Other chronic nonalcoholic liver disease | 4.03E-01 | 0.005 | 0.006 | 4.55E-01 | 0.002 | 0.003 | 4.03E-01 |
| 720 | Spinal stenosis | 4.11E-01 | 0.005 | 0.007 | 4.11E-01 | -0.001 | 0.003 | 6.47E-01 |
| 198.6 | Secondary malignancy of bone | 4.24E-01 | -0.012 | 0.032 | 7.11E-01 | 0.010 | 0.012 | 4.24E-01 |
| 594.1 | Calculus of kidney | 4.27E-01 | -0.005 | 0.006 | 4.27E-01 | -0.001 | 0.002 | 5.93E-01 |
| 271.3 | Intestinal disaccharidase deficiencies and disaccharide malabsorption | 4.35E-01 | 0.004 | 0.005 | 4.35E-01 | 0.001 | 0.002 | 6.58E-01 |
| 1100 | Family history | 4.43E-01 | 0.001 | 0.006 | 9.02E-01 | 0.002 | 0.002 | 4.43E-01 |
| 599.4 | Urinary incontinence | 4.52E-01 | 0.004 | 0.005 | 4.52E-01 | 0.000 | 0.002 | 8.77E-01 |
| 561.1 | Diarrhea | 4.53E-01 | 0.003 | 0.004 | 4.53E-01 | 0.000 | 0.002 | 8.36E-01 |
| 1005 | Other symptoms | 4.54E-01 | 0.004 | 0.005 | 4.54E-01 | -0.001 | 0.002 | 6.48E-01 |
| 300.12 | Agorophobia, social phobia, and panic disorder | 4.57E-01 | 0.005 | 0.008 | 5.54E-01 | -0.003 | 0.003 | 4.57E-01 |
| 745 | Pain in joint | 4.58E-01 | -0.001 | 0.003 | 7.62E-01 | -0.001 | 0.001 | 4.58E-01 |
| 574.1 | Cholelithiasis | 4.73E-01 | 0.000 | 0.007 | 9.96E-01 | -0.002 | 0.003 | 4.73E-01 |
| 426.31 | Right bundle branch block | 4.76E-01 | 0.001 | 0.009 | 9.23E-01 | 0.003 | 0.004 | 4.76E-01 |
| 535.8 | Other specified gastritis | 4.78E-01 | 0.003 | 0.009 | 7.57E-01 | -0.003 | 0.004 | 4.78E-01 |
| 008 | Intestinal infection | 4.87E-01 | 0.006 | 0.008 | 4.87E-01 | -0.002 | 0.004 | 5.59E-01 |
| 475 | Chronic sinusitis | 5.01E-01 | -0.001 | 0.005 | 8.30E-01 | -0.002 | 0.002 | 5.01E-01 |
| 278 | Overweight, obesity and other hyperalimentation | 5.02E-01 | -0.003 | 0.004 | 5.02E-01 | 0.001 | 0.002 | 5.23E-01 |
| 512.2 | Painful respiration | 5.14E-01 | -0.002 | 0.005 | 7.17E-01 | -0.001 | 0.002 | 5.14E-01 |
| 780 | Hypothermia/Chills | 5.15E-01 | 0.005 | 0.008 | 5.15E-01 | 0.000 | 0.003 | 9.48E-01 |
| 579.8 | Nonspecific abnormal findings in stool contents | 5.16E-01 | 0.004 | 0.006 | 5.16E-01 | 0.001 | 0.003 | 8.00E-01 |
| 705.8 | Hyperhidrosis | 5.18E-01 | 0.004 | 0.007 | 5.18E-01 | -0.002 | 0.003 | 5.47E-01 |
| 465 | Acute upper respiratory infections of multiple or unspecified sites | 5.28E-01 | -0.003 | 0.004 | 5.28E-01 | 0.000 | 0.002 | 8.40E-01 |
| 415.11 | Pulmonary embolism and infarction, acute | 5.48E-01 | 0.005 | 0.008 | 5.48E-01 | 0.000 | 0.004 | 9.54E-01 |
| 350.2 | Abnormality of gait | 5.49E-01 | 0.001 | 0.007 | 9.29E-01 | 0.002 | 0.003 | 5.49E-01 |
| 483 | Acute bronchitis and bronchiolitis | 5.54E-01 | -0.003 | 0.005 | 5.54E-01 | 0.000 | 0.002 | 9.18E-01 |
| 1019 | Other ill-defined and unknown causes of morbidity and mortality | 5.62E-01 | 0.004 | 0.007 | 5.62E-01 | -0.001 | 0.003 | 7.57E-01 |
| 290.1 | Dementias | 5.67E-01 | 0.005 | 0.009 | 5.67E-01 | 0.000 | 0.004 | 9.20E-01 |
| 741.4 | Joint effusions | 5.73E-01 | 0.003 | 0.005 | 5.73E-01 | 0.000 | 0.002 | 8.02E-01 |
| 426 | Cardiac conduction disorders | 5.77E-01 | 0.004 | 0.012 | 7.23E-01 | 0.003 | 0.005 | 5.77E-01 |
| 296 | Mood disorders | 5.81E-01 | 0.003 | 0.006 | 5.81E-01 | 0.001 | 0.003 | 7.85E-01 |
| 211 | Benign neoplasm of other parts of digestive system | 5.83E-01 | 0.002 | 0.009 | 7.87E-01 | 0.002 | 0.004 | 5.83E-01 |
| 433.31 | Transient cerebral ischemia | 5.85E-01 | -0.003 | 0.006 | 5.85E-01 | 0.001 | 0.003 | 6.03E-01 |
| 464 | Acute sinusitis | 6.00E-01 | -0.004 | 0.007 | 6.03E-01 | -0.002 | 0.003 | 6.00E-01 |
| 472 | Chronic pharyngitis and nasopharyngitis | 6.18E-01 | 0.002 | 0.006 | 7.41E-01 | 0.001 | 0.003 | 6.18E-01 |
| 292.1 | Aphasia/speech disturbance | 6.19E-01 | 0.004 | 0.008 | 6.19E-01 | 0.000 | 0.003 | 8.94E-01 |
| 202.2 | Non-Hodgkins lymphoma | 6.50E-01 | 0.001 | 0.012 | 9.07E-01 | -0.002 | 0.005 | 6.50E-01 |
| 535.9 | Gastritis and duodenitis, NOS | 6.61E-01 | -0.004 | 0.008 | 6.61E-01 | 0.001 | 0.003 | 6.70E-01 |
| 297.1 | Suicidal ideation | 6.71E-01 | 0.004 | 0.009 | 6.71E-01 | 0.000 | 0.004 | 9.99E-01 |
| 473.4 | Voice disturbance | 6.89E-01 | 0.002 | 0.007 | 8.15E-01 | 0.001 | 0.003 | 6.89E-01 |
| 1002 | Symptoms concerning nutrition, metabolism, and development | 6.94E-01 | -0.002 | 0.007 | 7.50E-01 | -0.001 | 0.003 | 6.94E-01 |
| 386.2 | Peripheral or central vertigo | 7.65E-01 | 0.002 | 0.010 | 8.72E-01 | -0.001 | 0.004 | 7.65E-01 |
| 740.11 | Osteoarthrosis, localized, primary | 7.73E-01 | 0.000 | 0.005 | 9.62E-01 | 0.001 | 0.002 | 7.73E-01 |
| 596.5 | Functional disorders of bladder | 7.90E-01 | 0.002 | 0.009 | 7.90E-01 | 0.000 | 0.004 | 9.25E-01 |
| 300.8 | Acute reaction to stress | 8.33E-01 | -0.002 | 0.009 | 8.33E-01 | 0.000 | 0.004 | 9.88E-01 |
| 272.13 | Mixed hyperlipidemia | 8.47E-01 | 0.000 | 0.005 | 9.86E-01 | 0.000 | 0.002 | 8.47E-01 |
| 366 | Cataract | 9.72E-01 | 0.000 | 0.007 | 9.72E-01 | 0.000 | 0.003 | 9.78E-01 |
